# Supplementary material for: Systematic analysis of RNA-binding proteins identifies targetable therapeutic vulnerabilities in osteosarcoma
Source: Nat Commun. 2024 Apr 1;15:2810. doi: 10.1038/s41467-024-47031-y (PMC10984982; doi:10.1038/s41467-024-47031-y)
Supplement: Supplementary file 1 — Supplementary Information [file 41467_2024_47031_MOESM1_ESM.pdf]

Supplementary Figure 1

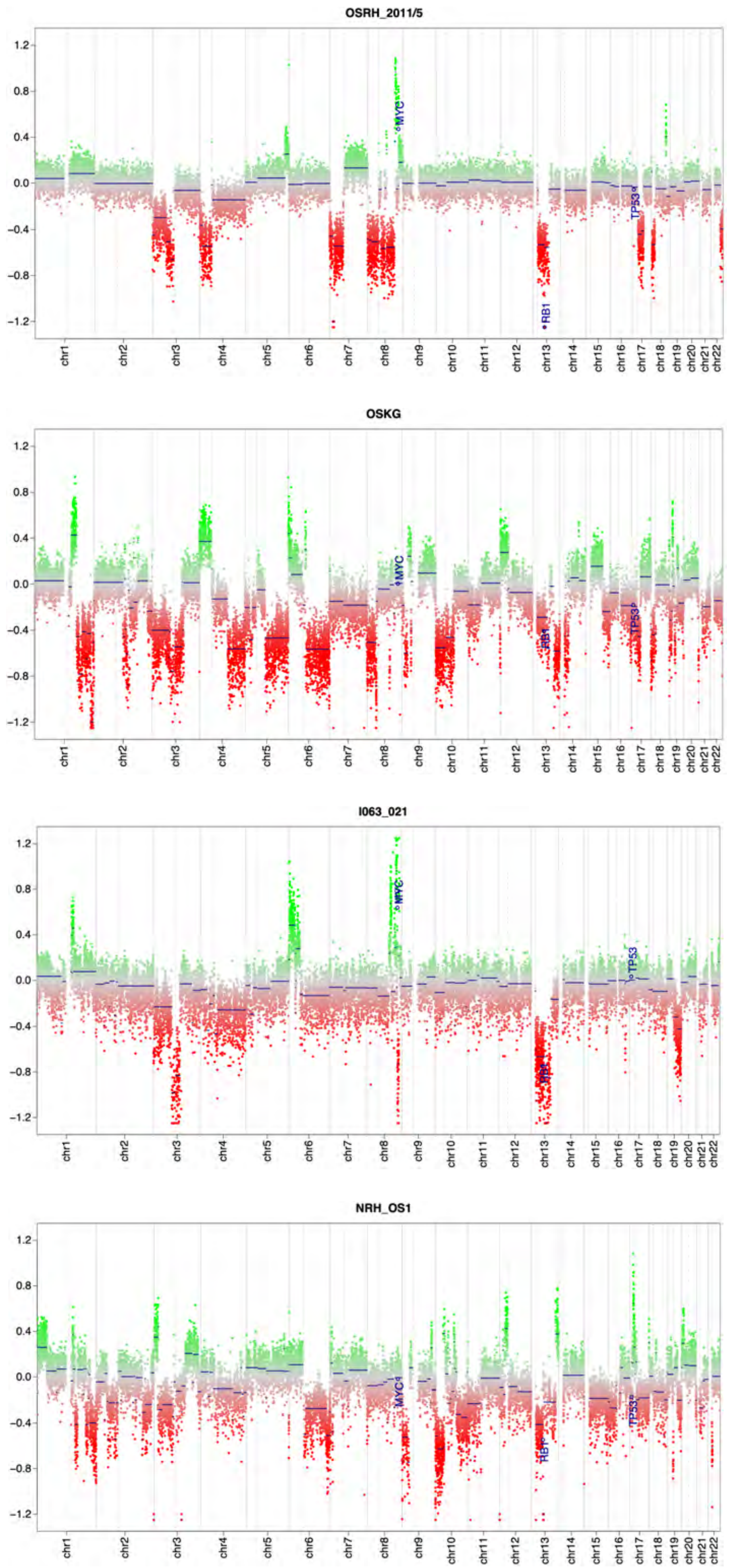

Supplementary Figure 1. Copy number variation profiles of primary patient-derived OS cells. Copy number gains/amplifications and losses are shown in green and red, respectively. Selected genes were labeled with gene names.

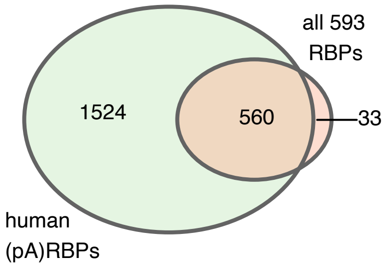

AHNAK2	FLNC	PAN3
ANXA6	GIGYF1	PAPD4
APOBEC3G	HN1L	PLS3
BIRC6	KPNA1	RNF214
COL1A1	L1RE1	SAMD9
COL1A2	LOC102724159	SETSIP
COL5A1	MAGED1	SMAD3
COL6A3	MTERF3	SMG7
DDX3Y	NAA16	STT3A
DSTN	OBSL1	TUBA1C
FASTK	PABPC5	TXLNA

Supplementary Figure 2. Venn diagrams showing the overlap of the RNA interactomes of the malignant and normal bone/mesenchymal studied here in comparison with previously identified RNA interactomes and RBPs with known RBDs. The datasets were collected from RBPbase (https://rbpbase.shiny.embl.de/).

Supplementary Figure 3

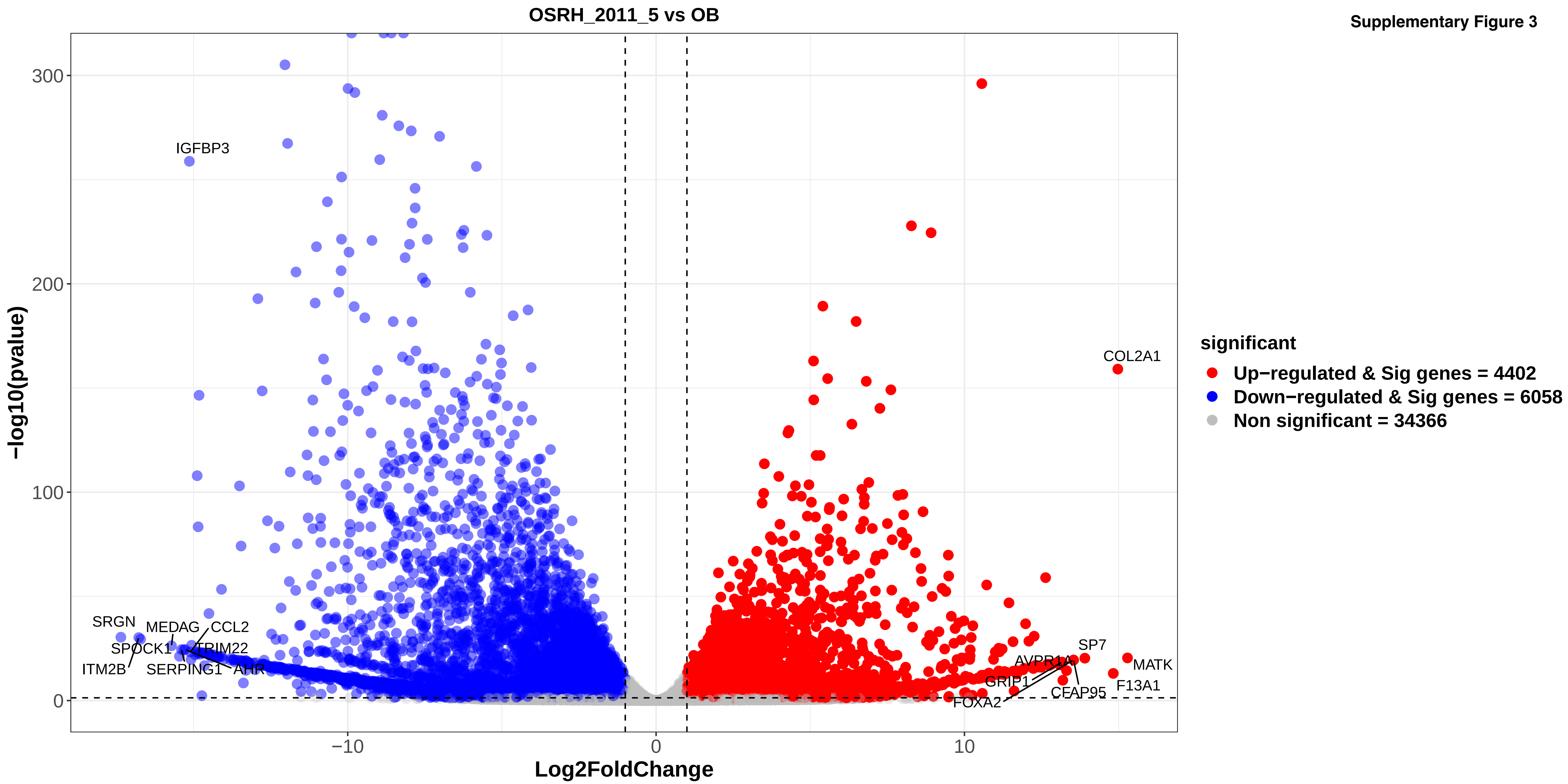

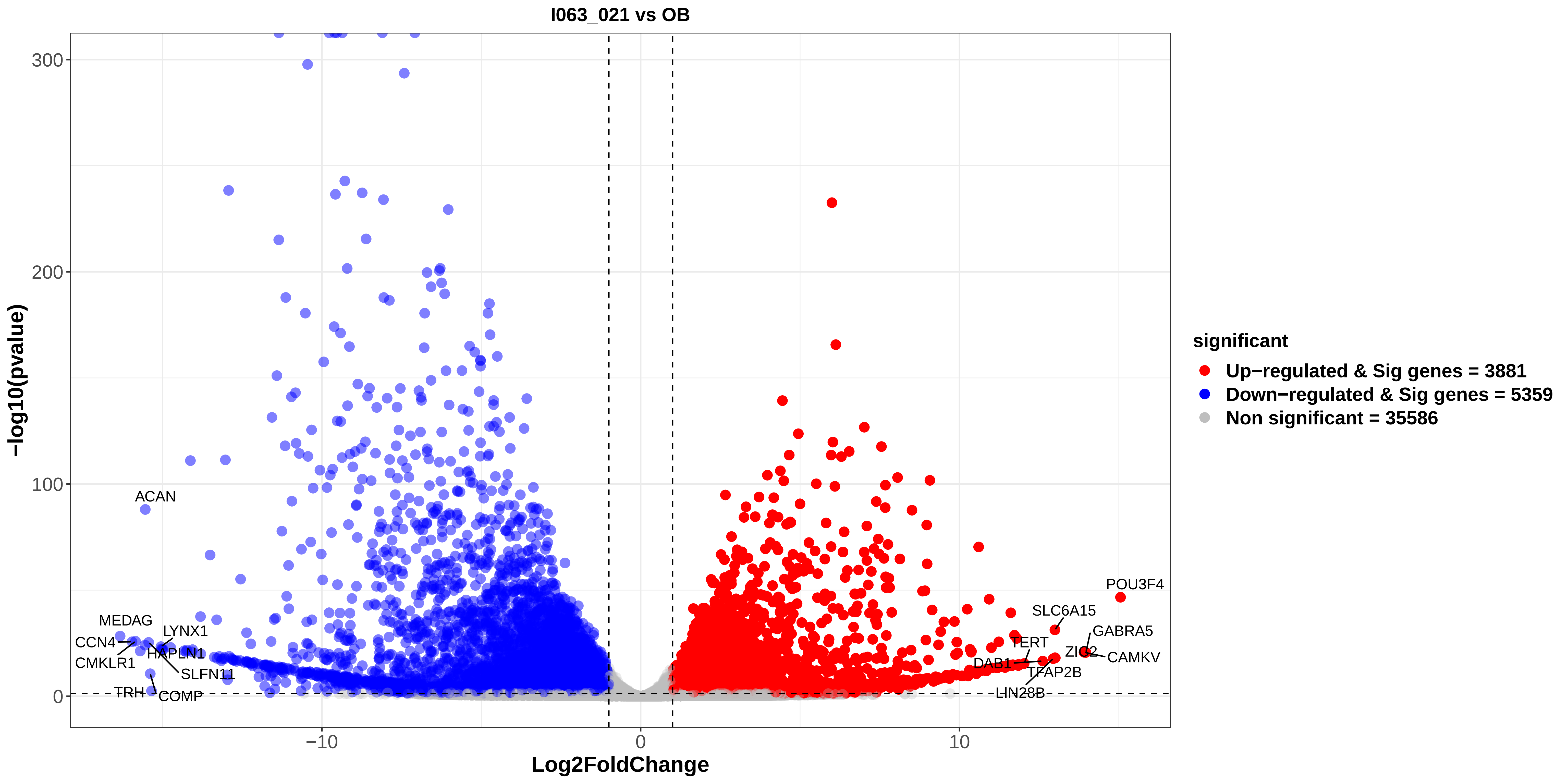
I063_021 vs OB
−log10(pvalue)
Log2FoldChange
300
200
100
0
−10
0
10
ACAN
MEDAG
LYNX1
CCN4
HAPLN1
CMKLR1
SLFN11
TRH
COMP
POU3F4
SLC6A15
GABRA5
TERT
ZIC2
CAMKV
DAB1
TFAP2B
LIN28B
significant
Up−regulated & Sig genes = 3881
Down−regulated & Sig genes = 5359
Non significant = 35586

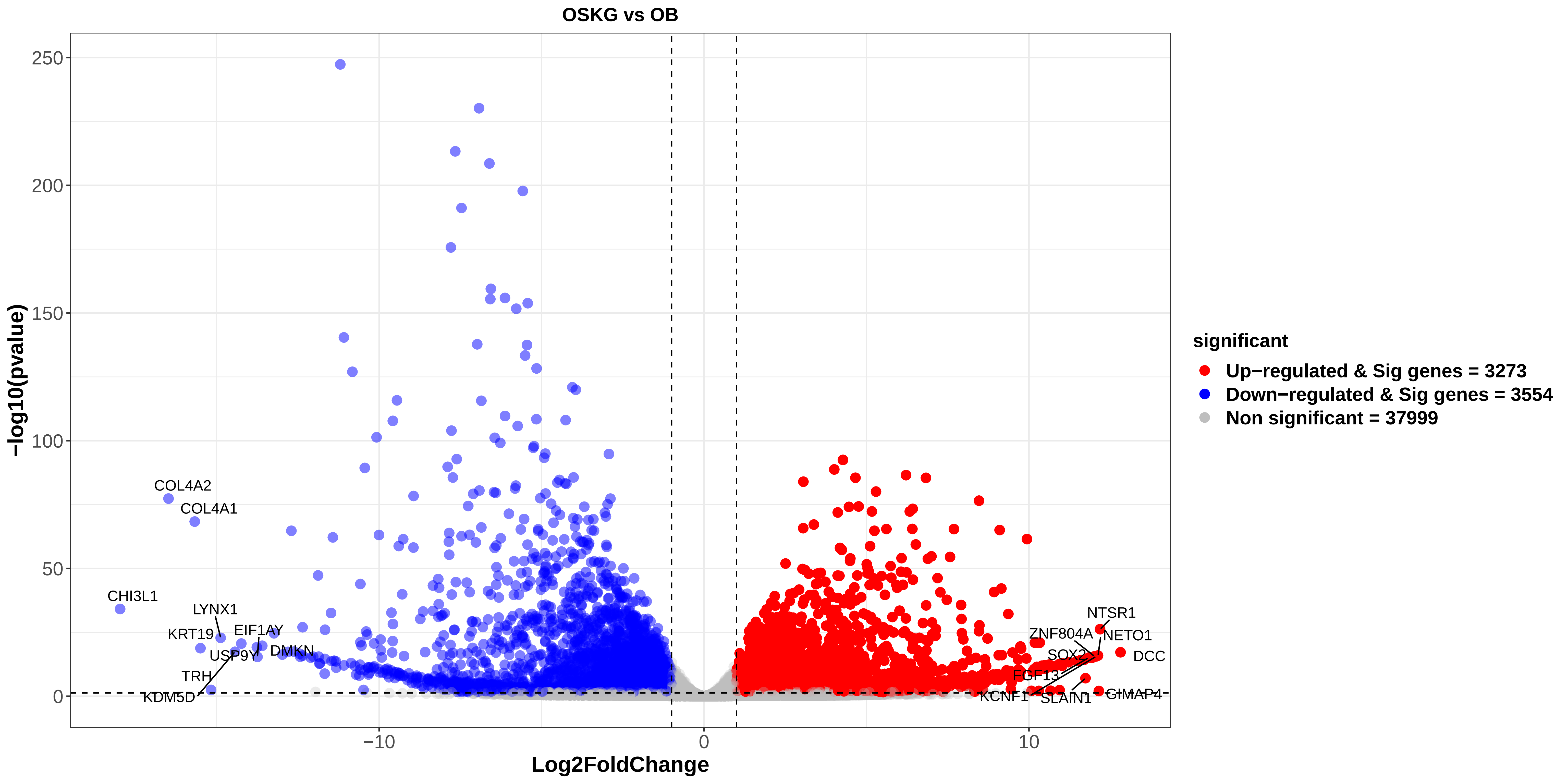
OSKG vs OB
-log10(pvalue)
Log2FoldChange
250
200
150
100
50
0
−10
0
10
COL4A2
COL4A1
CHI3L1
LYNX1
KRT19
EIF1AY
USP9Y
DMKN
TRH
KDM5D
NTSR1
ZNF804A
NETO1
SOX2
DCC
FGF13
KCNF1
SLAIN1
GIMAP4
significant
Up−regulated & Sig genes = 3273
Down−regulated & Sig genes = 3554
Non significant = 37999

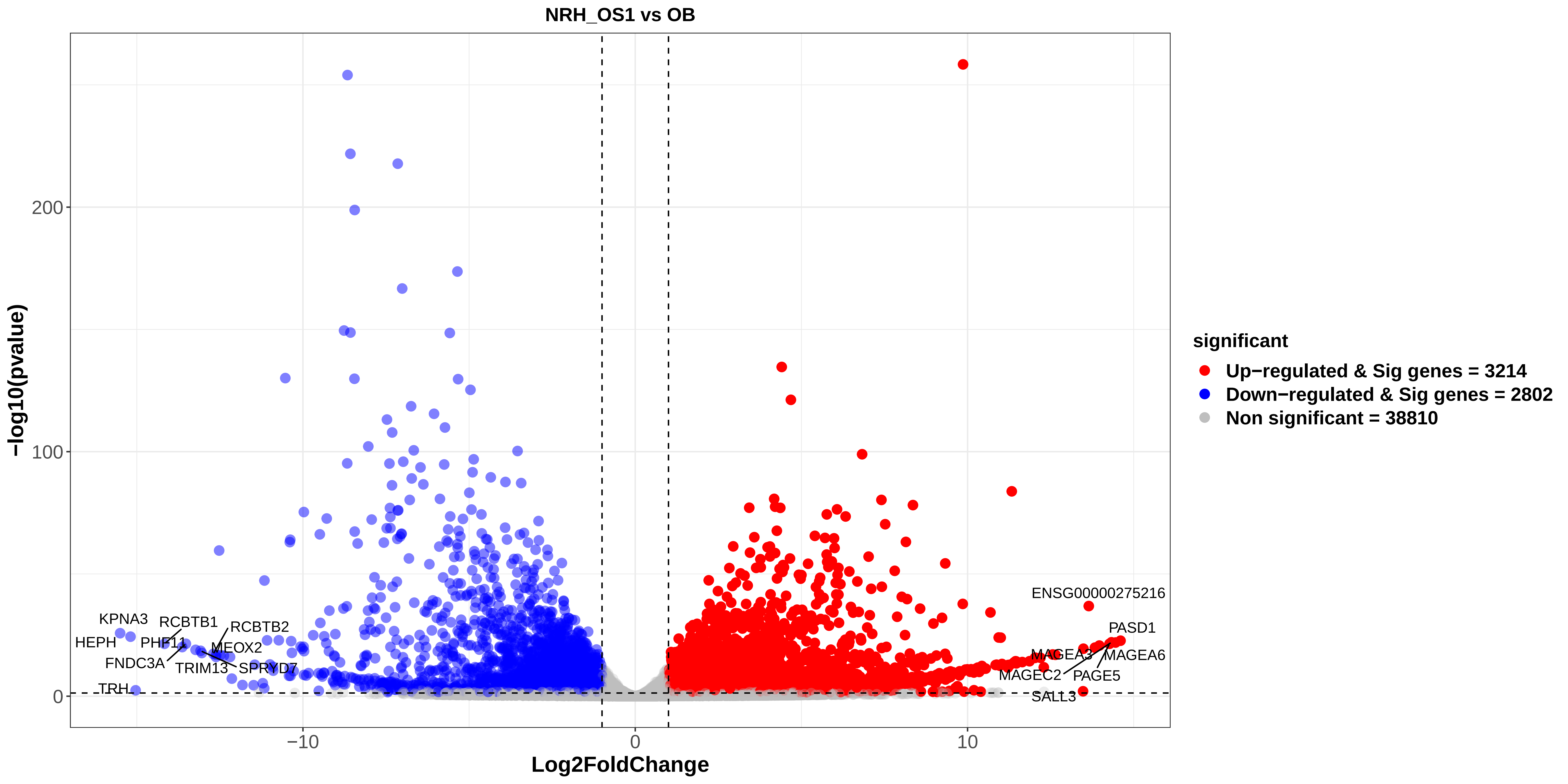
NRH_OS1 vs OB
significant
Up−regulated & Sig genes = 3214
Down−regulated & Sig genes = 2802
Non significant = 38810
−log10(pvalue)
Log2FoldChange
200
100
0
−10
0
10
KPNA3
HEPH
RCBTB1
PHF11
FNDC3A
RCBTB2
MEOX2
TRIM13
SPRYD7
TRH
ENSG00000275216
PASD1
MAGEA3
MAGEA6
MAGEC2
PAGE5
SALL3

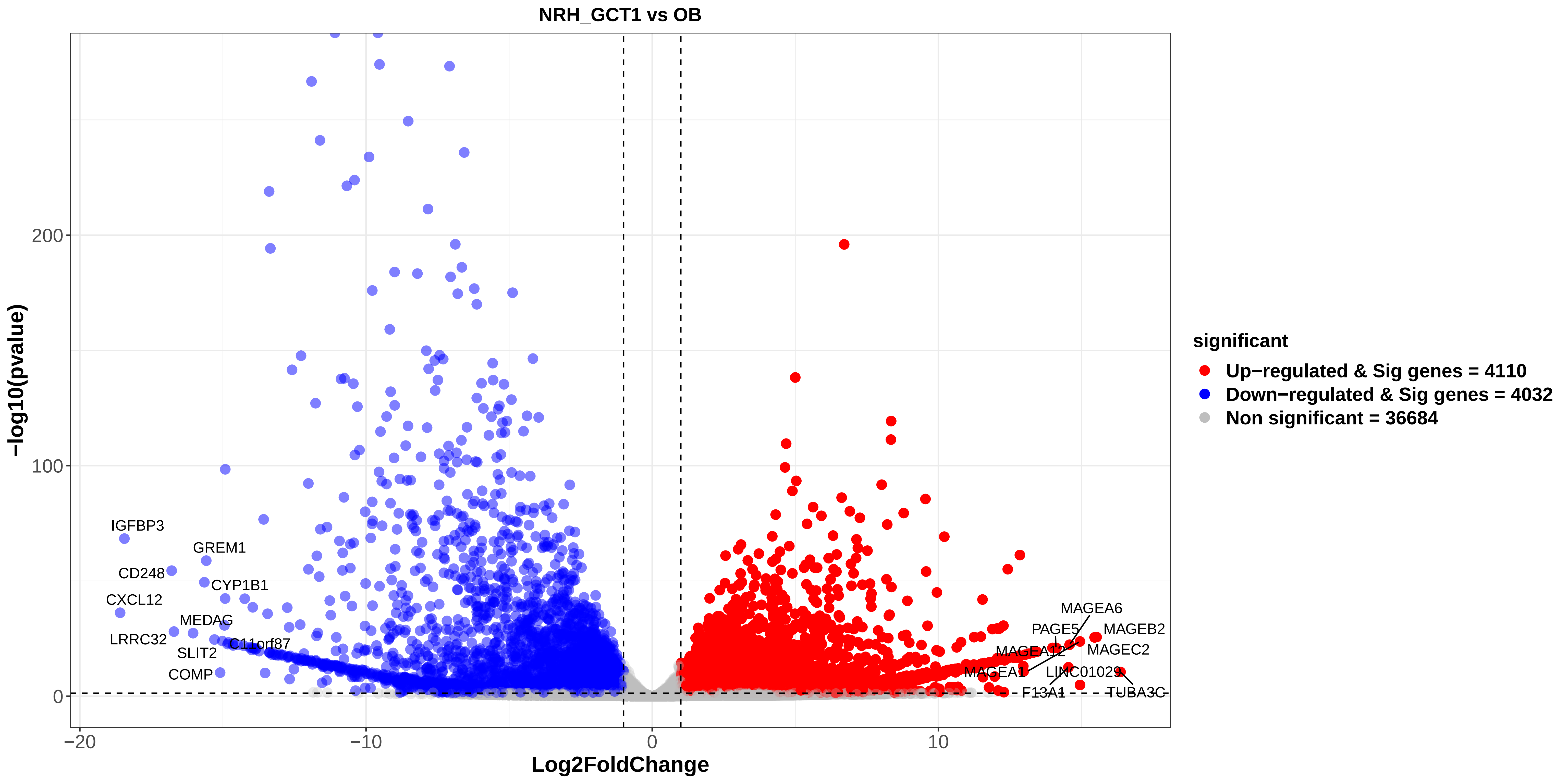

NRH_GCT1 vs OB
−log10(pvalue)
Log2FoldChange
200
100
0
−20
−10
0
10
IGFBP3
GREM1
CD248
CYP1B1
CXCL12
MEDAG
LRRC32
C11orf87
SLIT2
COMP
MAGEA6
PAGE5
MAGEB2
MAGEA12
MAGEC2
MAGEA1
LINC01029
F13A1
TUBA3C
significant
Up−regulated & Sig genes = 4110
Down−regulated & Sig genes = 4032
Non significant = 36684

Supplementary Figure 3. Volcano plot showing the differentially expressed genes in the transcriptomes of OS and GCTB cells compared to OB cells determined by the DESeq2 tool. Each dot represents a gene; red dots denote the genes that are significantly up-regulated (log2FC > 1 & padj < 0.05); blue dots denote the genes that are significantly down-regulated (log2FC < -1 & padj < 0.05); grey dots denote the non-significant genes. The X-axis represents log2foldChange and Y-axis represents the pvalue in -log10scale.

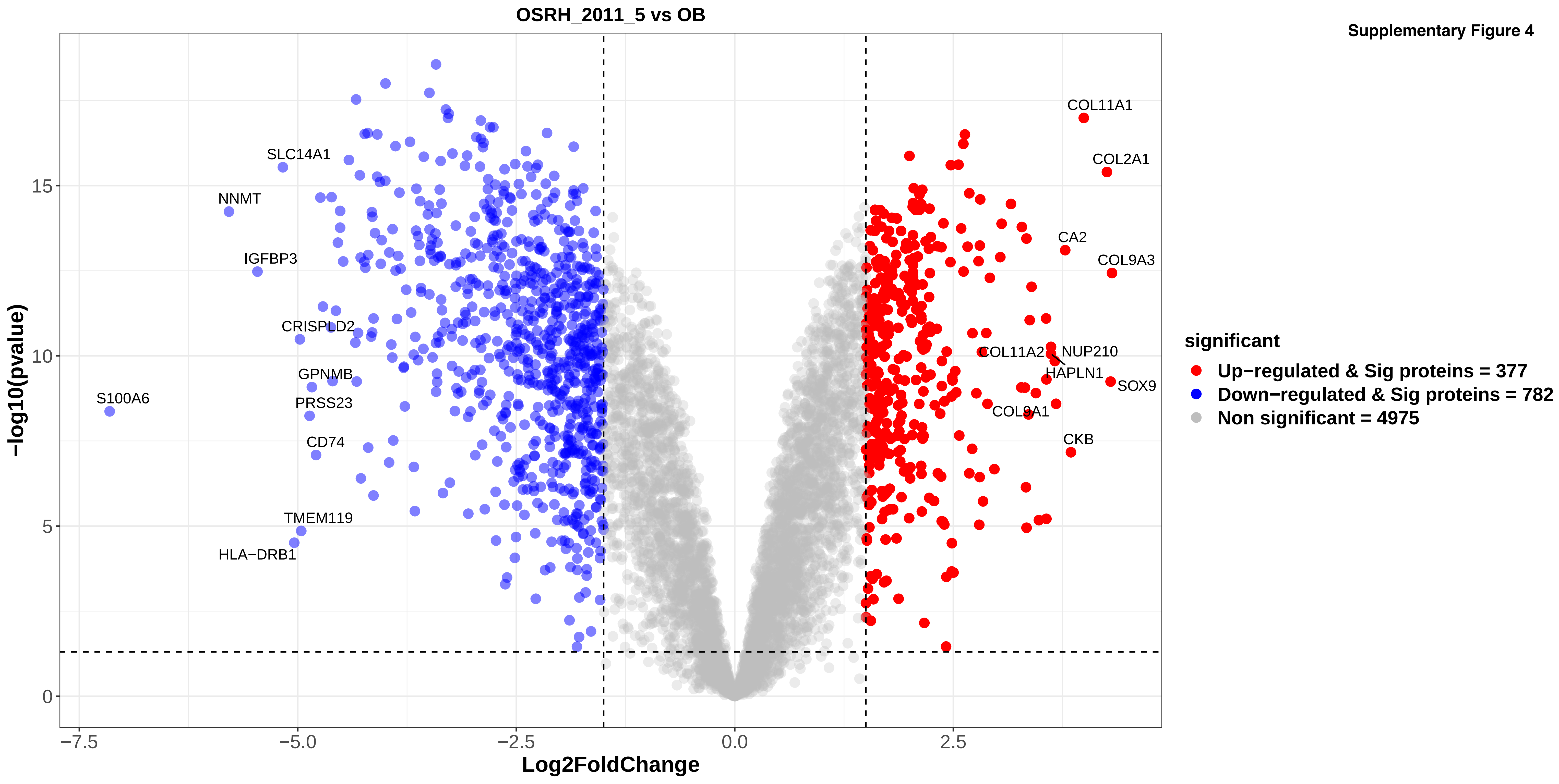
OSRH_2011_5 vs OB
COL11A1
COL2A1
CA2
COL9A3
COL11A2
NUP210
HAPLN1
SOX9
COL9A1
CKB
SLC14A1
NNMT
IGFBP3
CRISPLD2
GPNMB
S100A6
PRSS23
CD74
TMEM119
HLA−DRB1
−log10(pvalue)
15
10
5
0
−7.5
−5.0
−2.5
0.0
2.5
Log2FoldChange
significant
Up−regulated & Sig proteins = 377
Down−regulated & Sig proteins = 782
Non significant = 4975

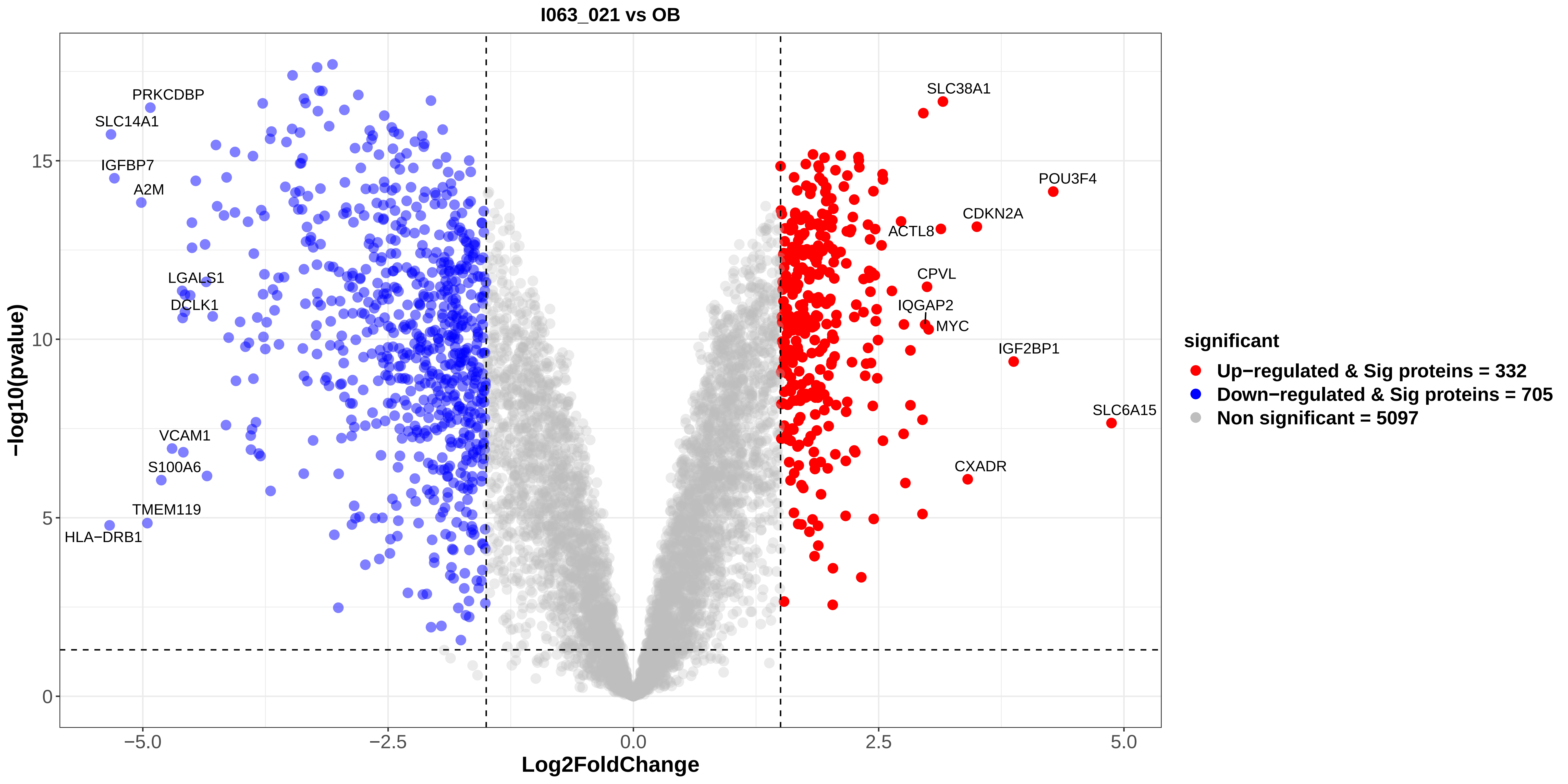
I063_021 vs OB
SLC38A1
PRKCDBP
SLC14A1
IGFBP7
A2M
POU3F4
CDKN2A
ACTL8
LGALS1
CPVL
DCLK1
IQGAP2
MYC
IGF2BP1
SLC6A15
VCAM1
S100A6
CXADR
TMEM119
HLA−DRB1
−log10(pvalue)
Log2FoldChange
15
10
5
0
−5.0
−2.5
0.0
2.5
5.0
significant
Up−regulated & Sig proteins = 332
Down−regulated & Sig proteins = 705
Non significant = 5097

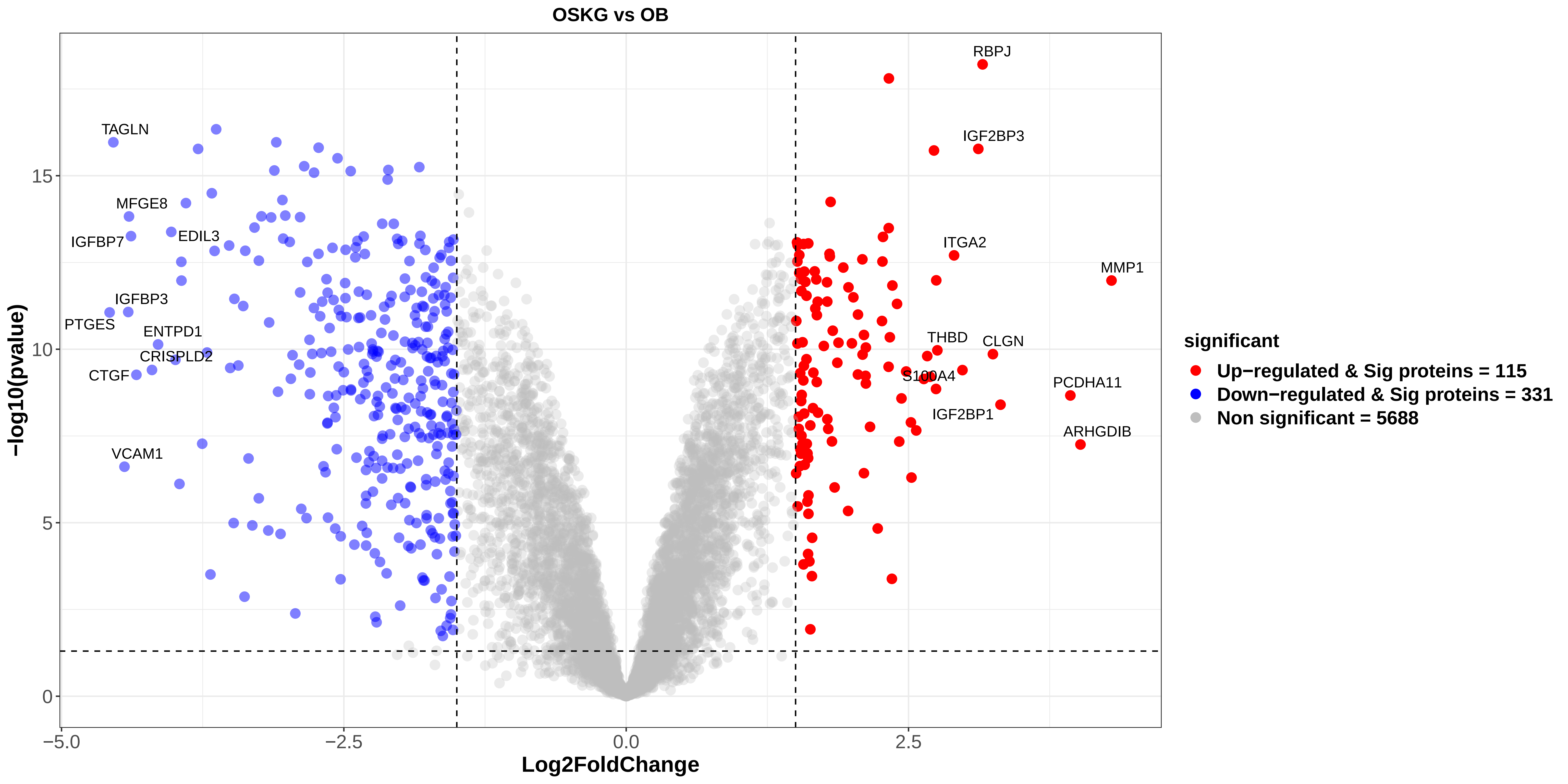

OSKG vs OB
RBPJ
IGF2BP3
TAGLN
MFGE8
EDIL3
IGFBP7
ITGA2
MMP1
IGFBP3
PTGES
ENTPD1
CRISPLD2
CTGF
THBD
CLGN
S100A4
PCDHA11
IGF2BP1
ARHGDIB
VCAM1
−log10(pvalue)
Log2FoldChange
15
10
5
0
−5.0
−2.5
0.0
2.5
significant
Up−regulated & Sig proteins = 115
Down−regulated & Sig proteins = 331
Non significant = 5688

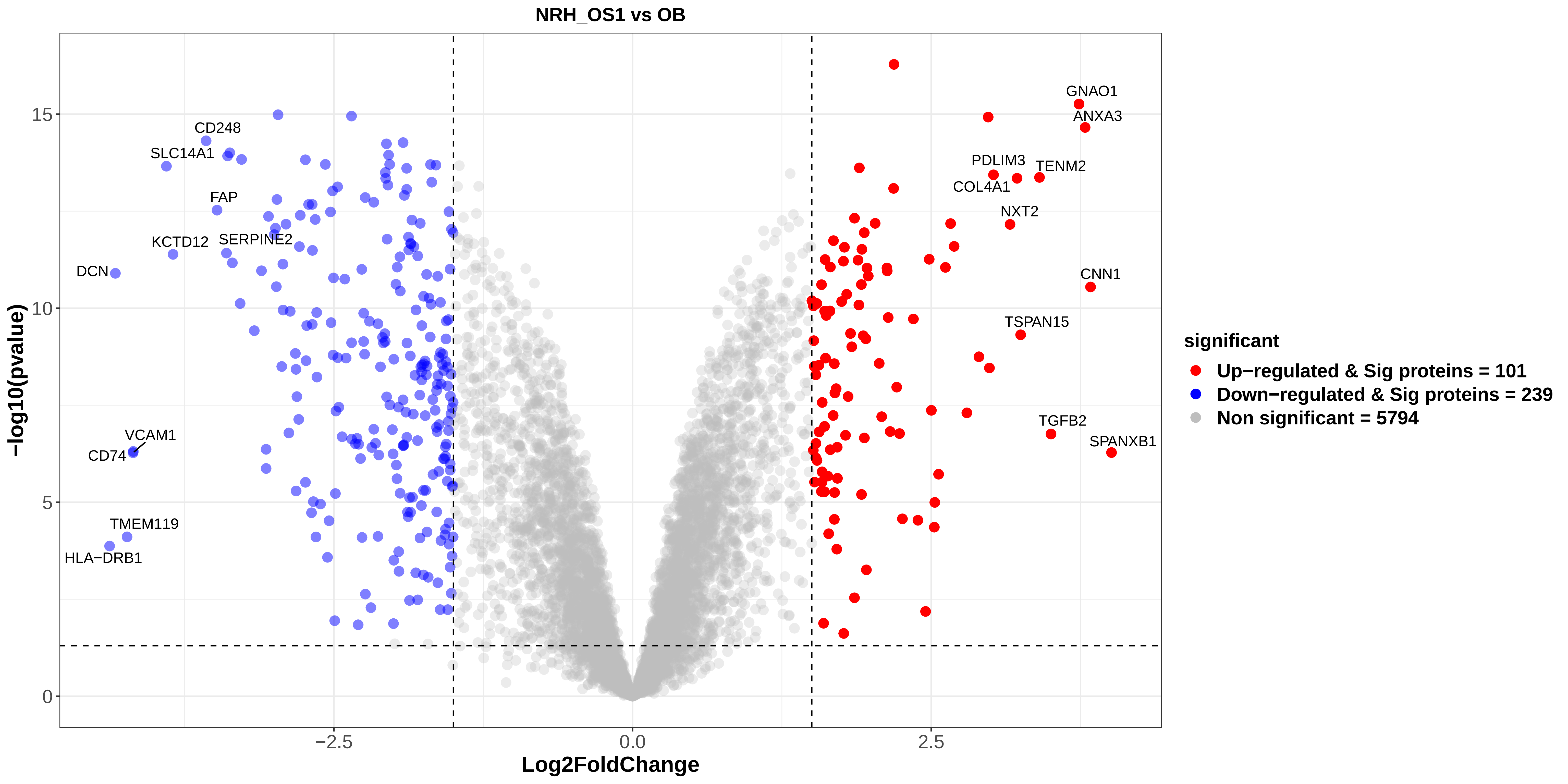

NRH_OS1 vs OB
GNAO1
ANXA3
CD248
SLC14A1
PDLIM3
TENM2
COL4A1
FAP
NXT2
KCTD12
SERPINE2
DCN
CNN1
TSPAN15
VCAM1
CD74
TGFB2
SPANXB1
TMEM119
HLA−DRB1
15
10
5
0
−2.5
0.0
2.5
−log10(pvalue)
Log2FoldChange
significant
Up−regulated & Sig proteins = 101
Down−regulated & Sig proteins = 239
Non significant = 5794

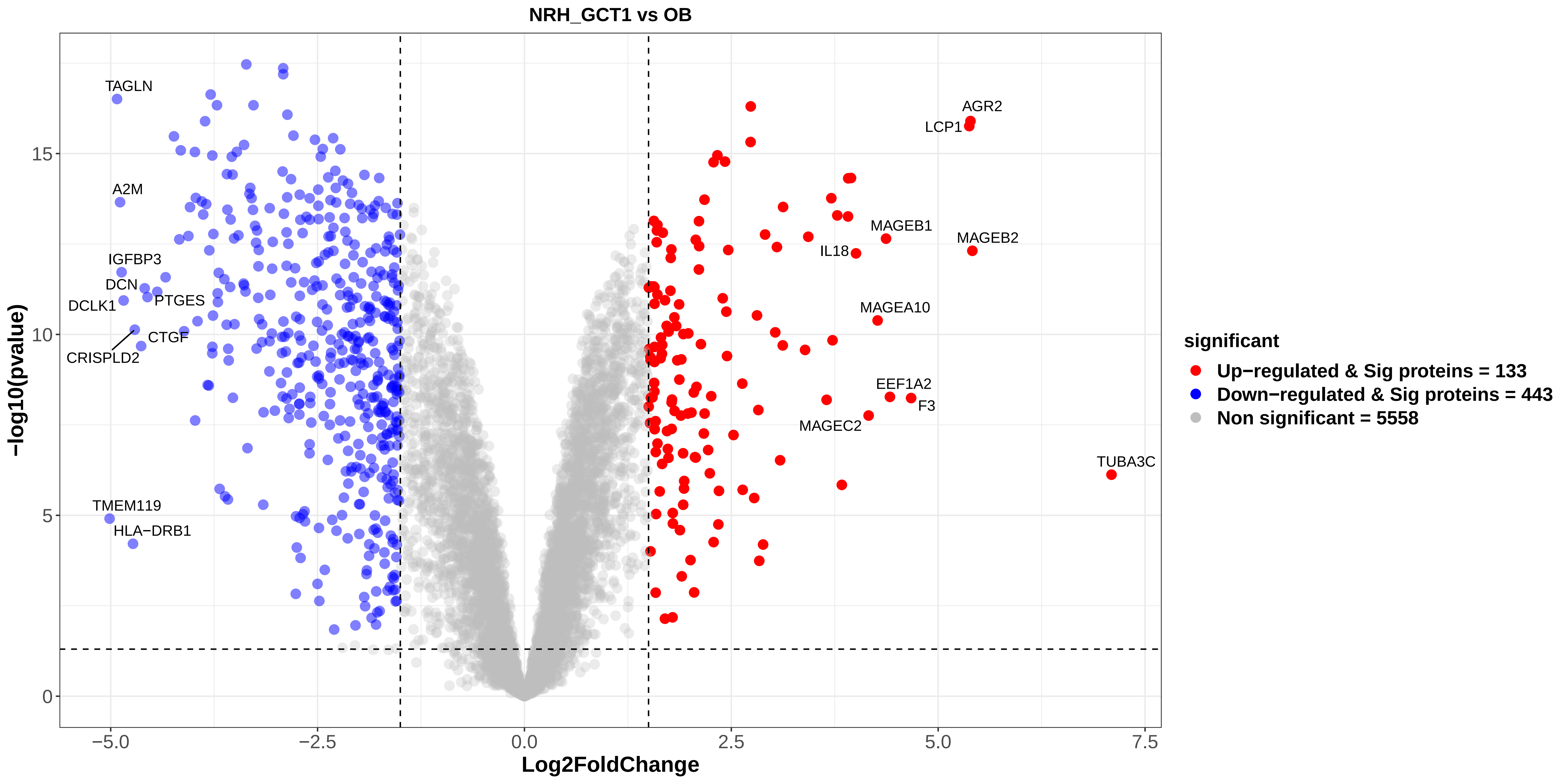

NRH_GCT1 vs OB
−log10(pvalue)
Log2FoldChange
15
10
5
0
−5.0
−2.5
0.0
2.5
5.0
7.5
TAGLN
A2M
IGFBP3
DCN
DCLK1
PTGES
CTGF
CRISPLD2
TMEM119
HLA−DRB1
AGR2
LCP1
MAGEB1
MAGEB2
IL18
MAGEA10
EEF1A2
F3
MAGEC2
TUBA3C
significant
Up−regulated & Sig proteins = 133
Down−regulated & Sig proteins = 443
Non significant = 5558

Supplementary Figure 4. Volcano plot showing the differentially expressed proteins in the proteomes of OS and GCTB cells compared to OB cells determined by the limma package. Each dot represents a protein; red dots denote the proteins that are significantly up-regulated (log2FC > 1.5 & padj < 0.05); blue dots denote the proteins that are significantly down-regulated (log2FC < -1.5 & padj < 0.05); grey dots denote the non-significant genes. The X-axis represents log2foldChange and Y-axis represents the pvalue in -log10scale.

Supplementary Figure 5

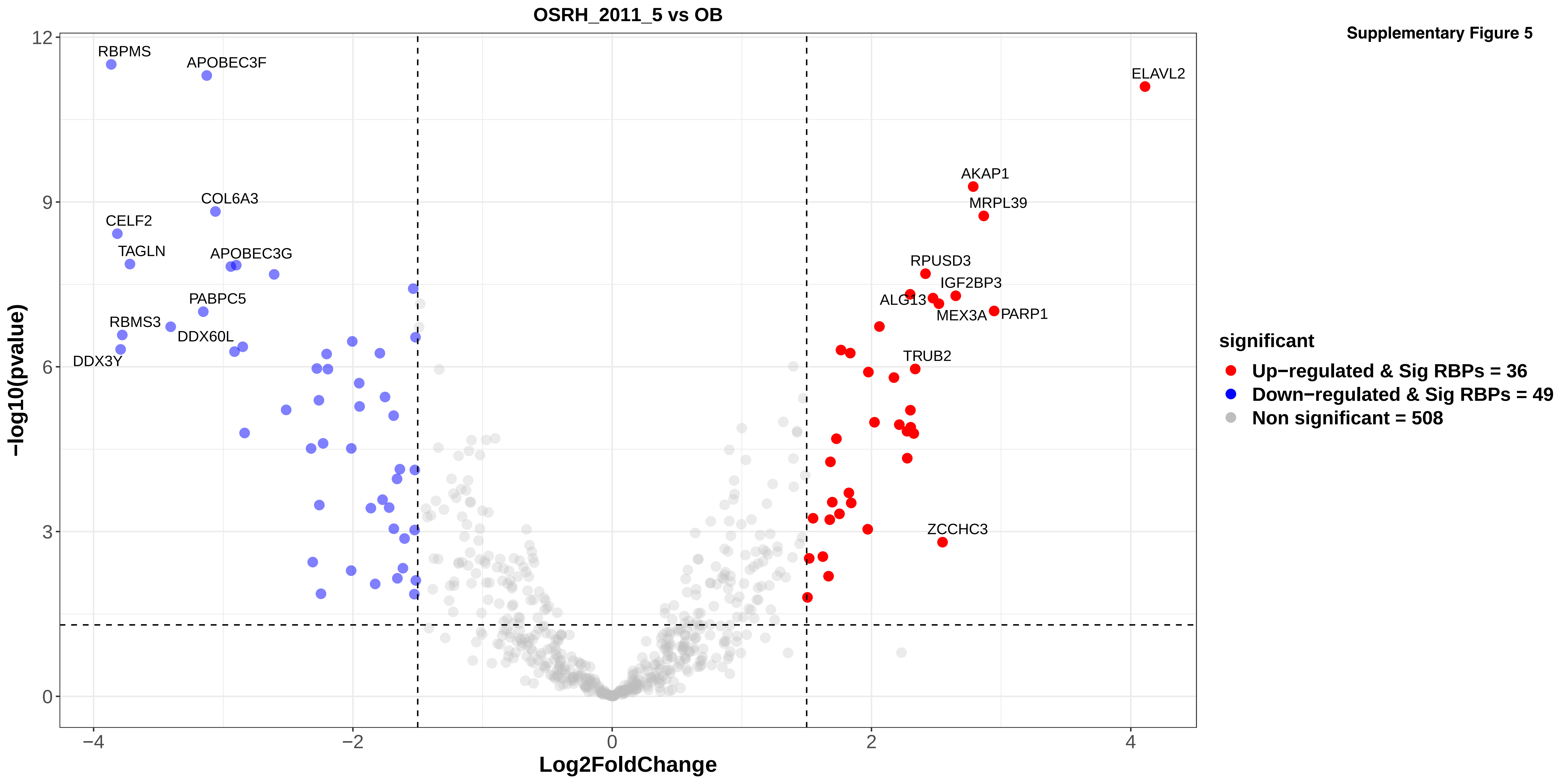

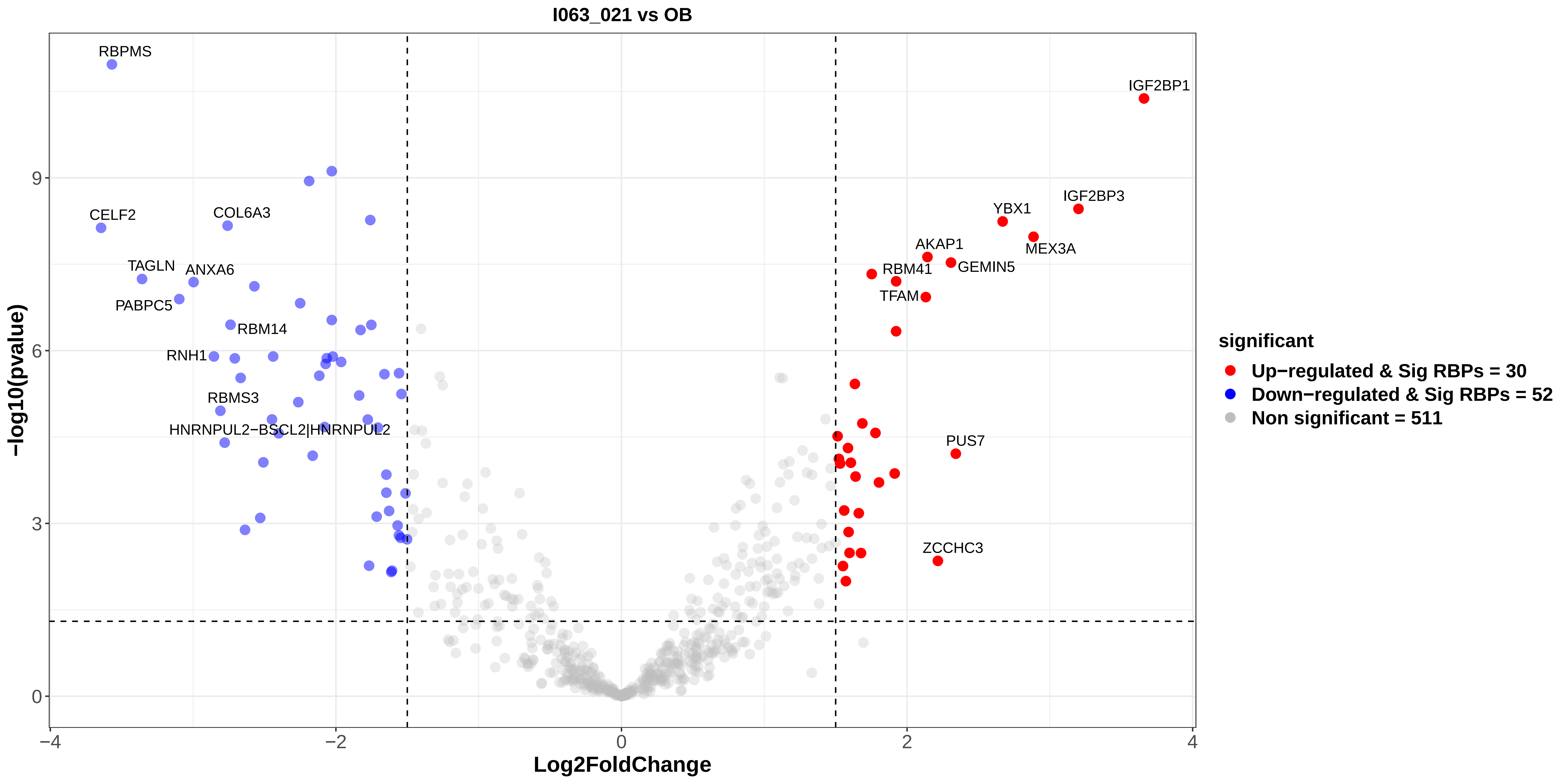
I063_021 vs OB
RBPMS
IGF2BP1
CELF2
COL6A3
YBX1
IGF2BP3
MEX3A
AKAP1
GEMIN5
RBM41
TAGLN
ANXA6
TFAM
PABPC5
RBM14
RNH1
RBMS3
HNRNPUL2−BSCL2|HNRNPUL2
PUS7
ZCCHC3
−log10(pvalue)
Log2FoldChange
9
6
3
0
−4
−2
0
2
4
significant
Up−regulated & Sig RBPs = 30
Down−regulated & Sig RBPs = 52
Non significant = 511

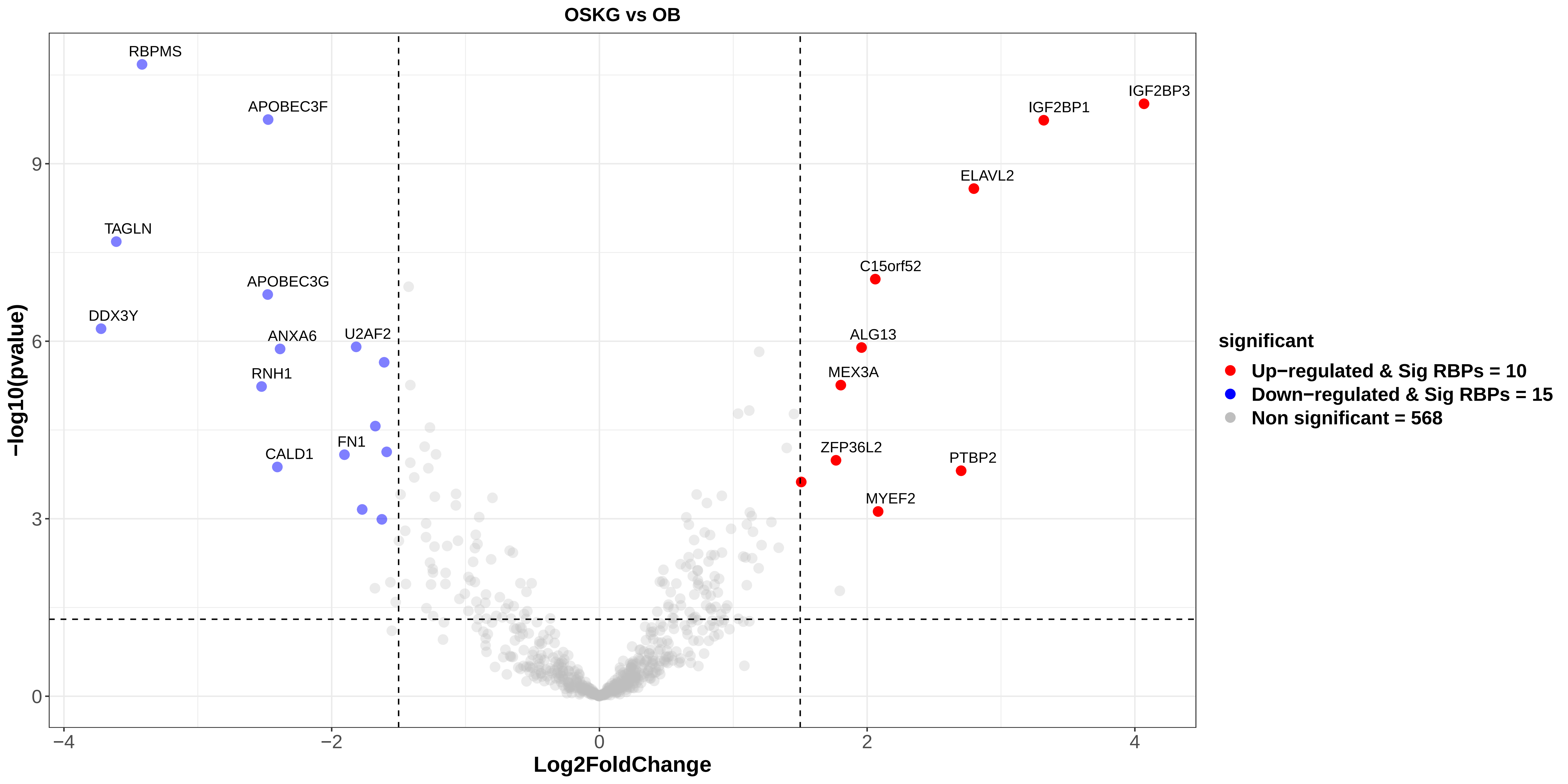
OSKG vs OB
RBPMS
APOBEC3F
TAGLN
APOBEC3G
DDX3Y
ANXA6
U2AF2
RNH1
FN1
CALD1
IGF2BP3
IGF2BP1
ELAVL2
C15orf52
ALG13
MEX3A
ZFP36L2
PTBP2
MYEF2
−log10(pvalue)
Log2FoldChange
9
6
3
0
−4
−2
0
2
4
significant
Up−regulated & Sig RBPs = 10
Down−regulated & Sig RBPs = 15
Non significant = 568

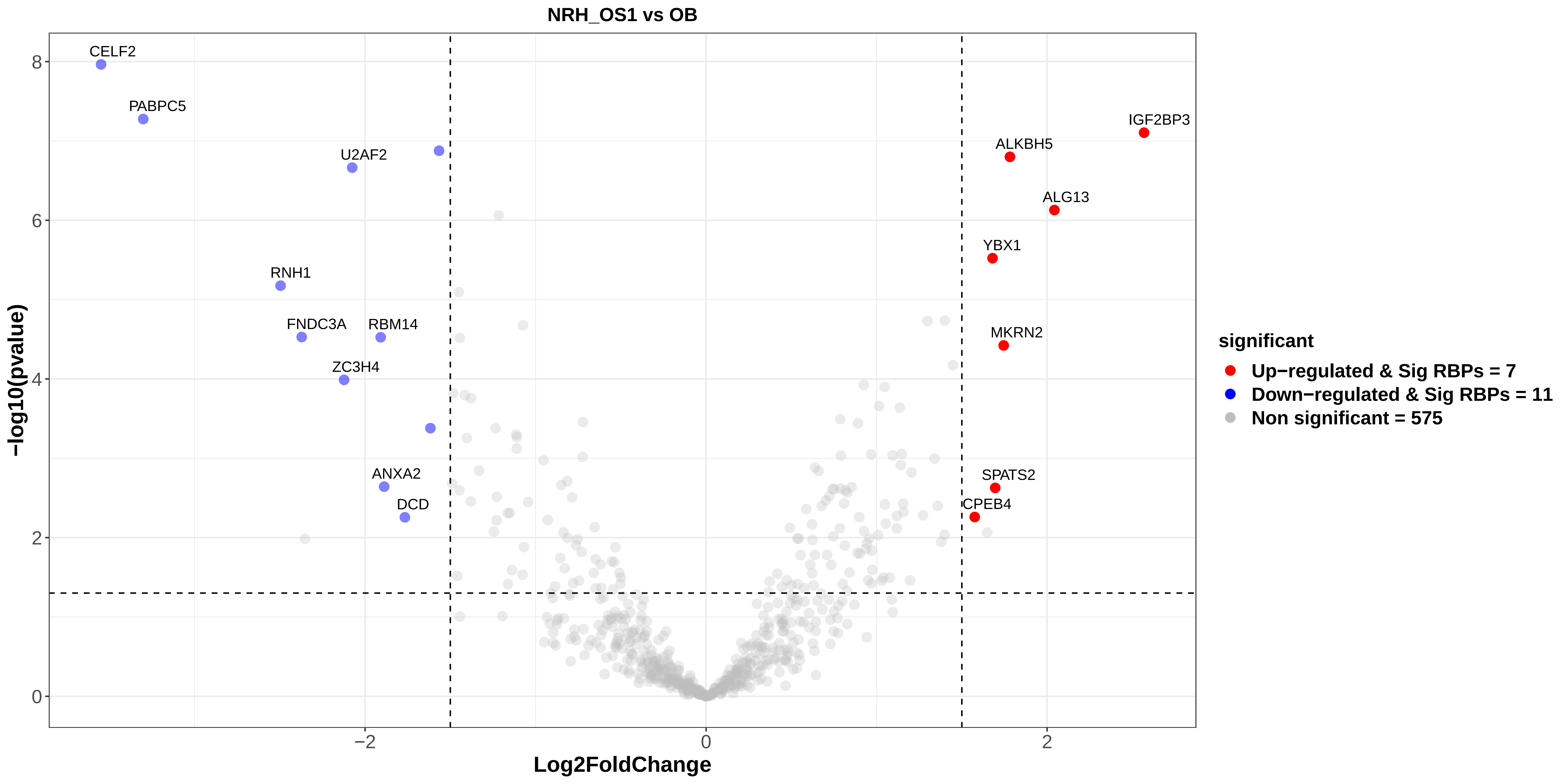
NRH_OS1 vs OB
CELF2
PABPC5
U2AF2
RNH1
FNDC3A
RBM14
ZC3H4
ANXA2
DCD
IGF2BP3
ALKBH5
ALG13
YBX1
MKRN2
SPATS2
CPEB4
−log10(pvalue)
Log2FoldChange
8
6
4
2
0
−2
0
2
significant
Up−regulated & Sig RBPs = 7
Down−regulated & Sig RBPs = 11
Non significant = 575

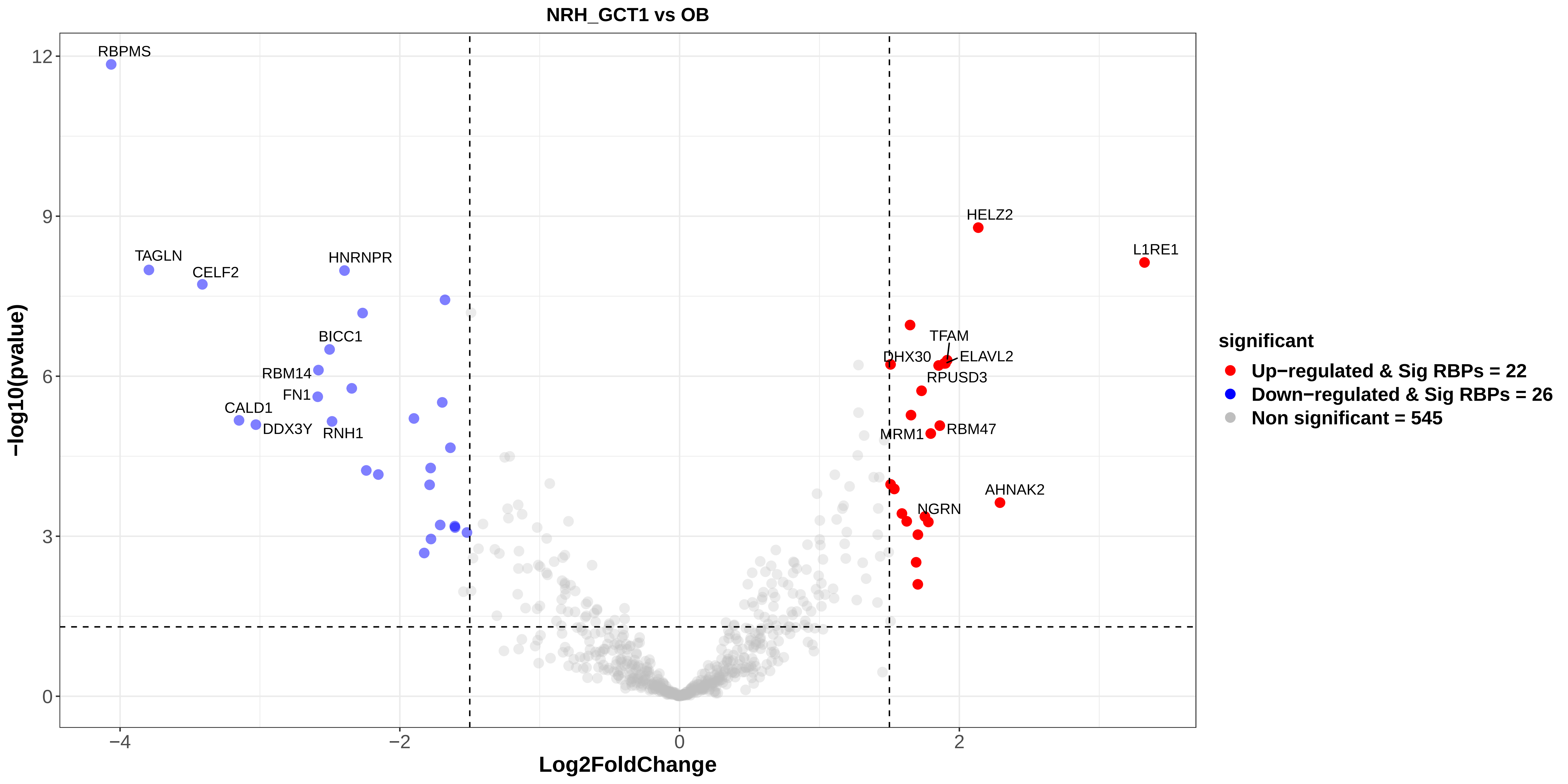

NRH_GCT1 vs OB
RBPMS
TAGLN
CELF2
HNRNPR
BICC1
RBM14
FN1
CALD1
DDX3Y
RNH1
HELZ2
L1RE1
TFAM
DHX30
ELAVL2
RPUSD3
MRM1
RBM47
AHNAK2
NGRN
−log10(pvalue)
Log2FoldChange
12
9
6
3
0
−4
−2
0
2
significant
Up−regulated & Sig RBPs = 22
Down−regulated & Sig RBPs = 26
Non significant = 545

Supplementary Figure 5. Volcano plot showing the differentially expressed RBPs in the RNA interactomes of OS and GCTB cells compared to OB cells determined by the limma package. Each dot represents a protein; red dots denote the RBPs that are significantly up-regulated (log2FC > 1.5 & padj < 0.05); blue dots denote the RBPs that are significantly down-regulated (log2FC < -1.5 & padj < 0.05); grey dots denote the non-significant genes. The X-axis represents log2foldChange and Y-axis represents the pvalue in -log10 scale.

Supplementary Figure 6

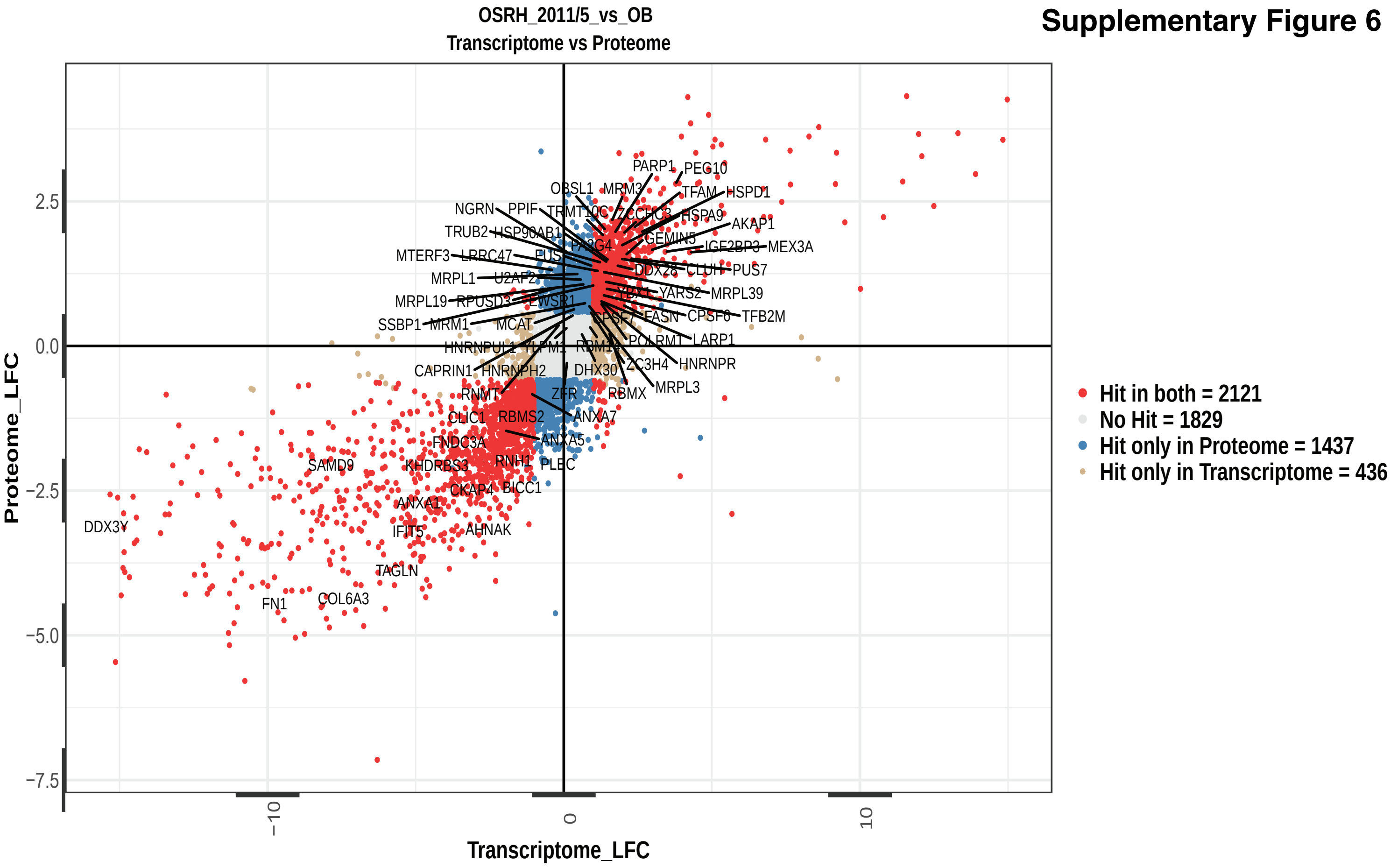

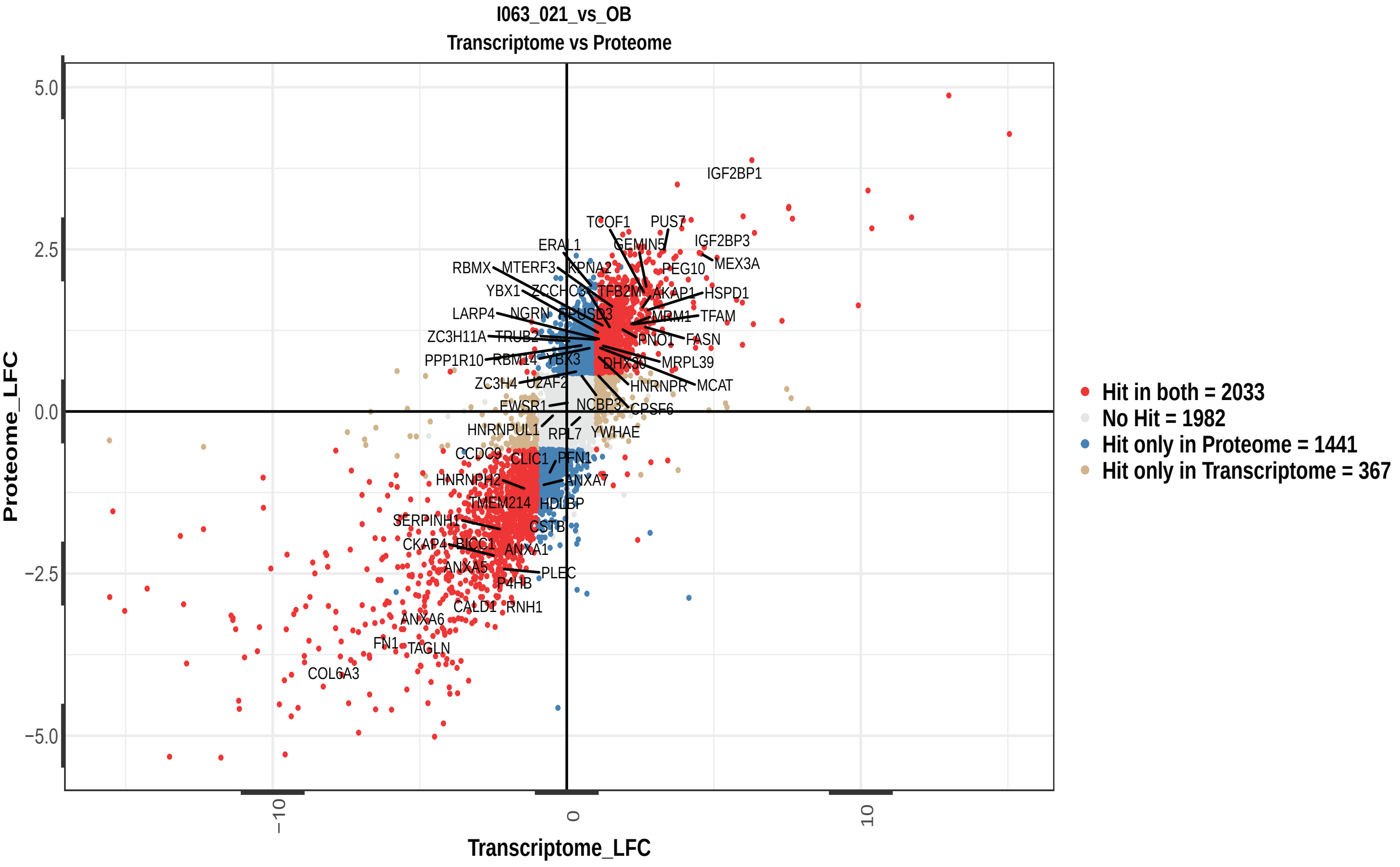

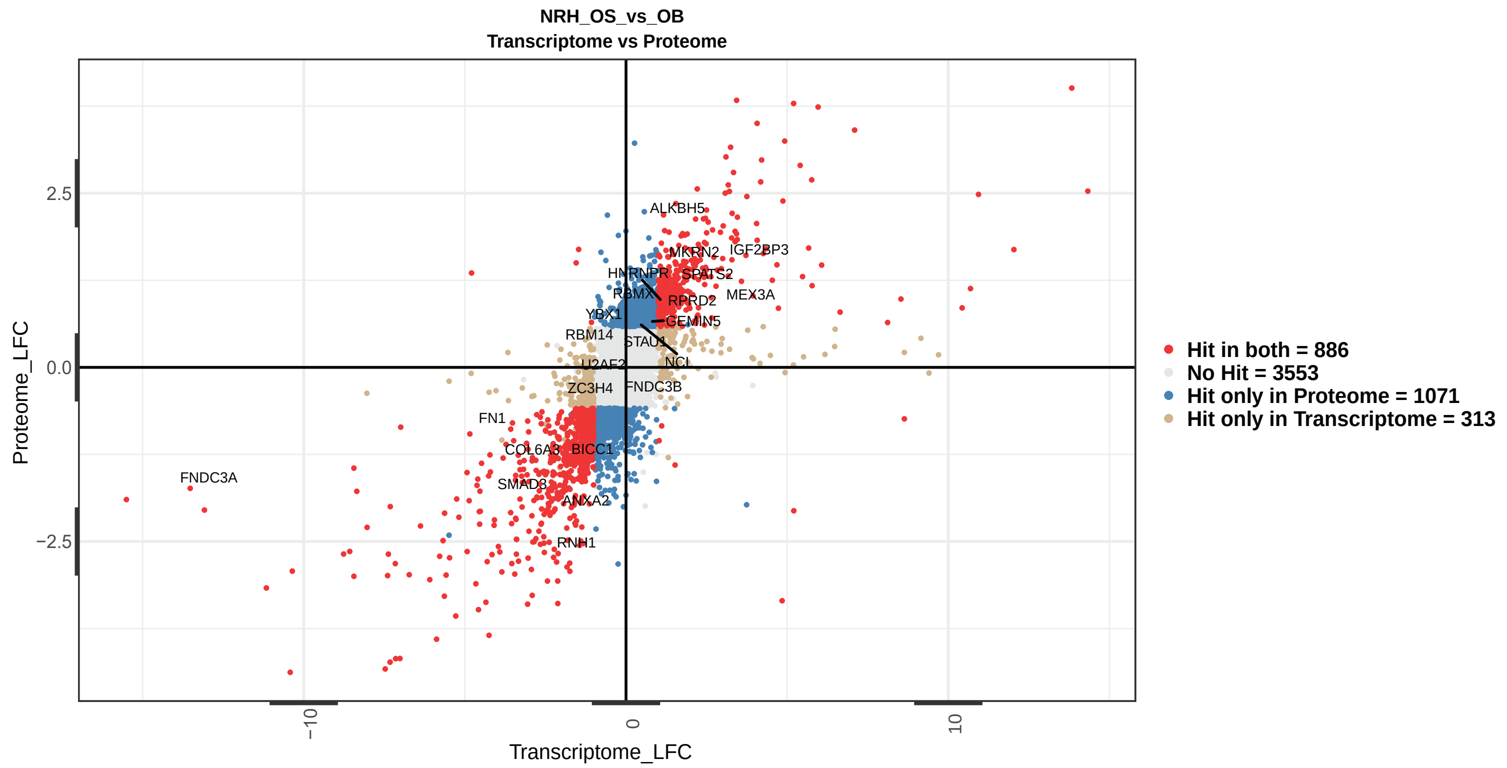
NRH_OS_vs_OB
Transcriptome vs Proteome
Proteome_LFC
Transcriptome_LFC
2.5
0.0
−2.5
−10
0
10
ALKBH5
MKRN2
IGF2BP3
HNRNPR
SPATS2
RBMX
RPRD2
MEX3A
YBX1
GEMIN5
RBM14
STAU1
NCL
U2AF2
ZC3H4
FNDC3B
FN1
COL6A3
BICC1
FNDC3A
SMAD3
ANXA2
RNH1
Hit in both = 886
No Hit = 3553
Hit only in Proteome = 1071
Hit only in Transcriptome = 313

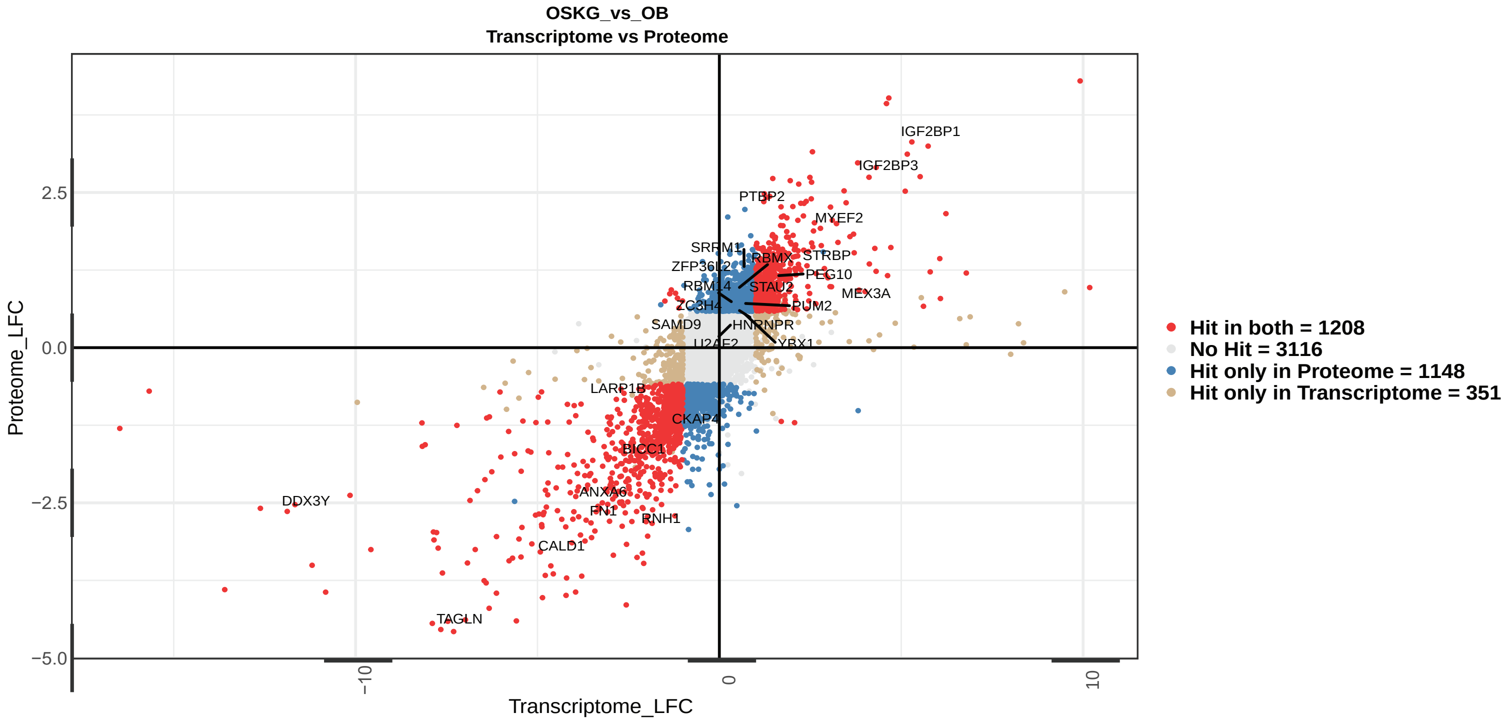
OSKG_vs_OB
Transcriptome vs Proteome
Proteome_LFC
Transcriptome_LFC
2.5
0.0
−2.5
−5.0
−10
0
10
IGF2BP1
IGF2BP3
PTBP2
MYEF2
SRRM1
RBMX
STRBP
ZFP36L2
PEG10
RBM14
STAU2
MEX3A
ZC3H4
PUM2
SAMD9
HNRNPR
U2AF2
YBX1
LARP1B
CKAP4
BICC1
ANXA6
FN1
RNH1
DDX3Y
CALD1
TAGLN
Hit in both = 1208
No Hit = 3116
Hit only in Proteome = 1148
Hit only in Transcriptome = 351

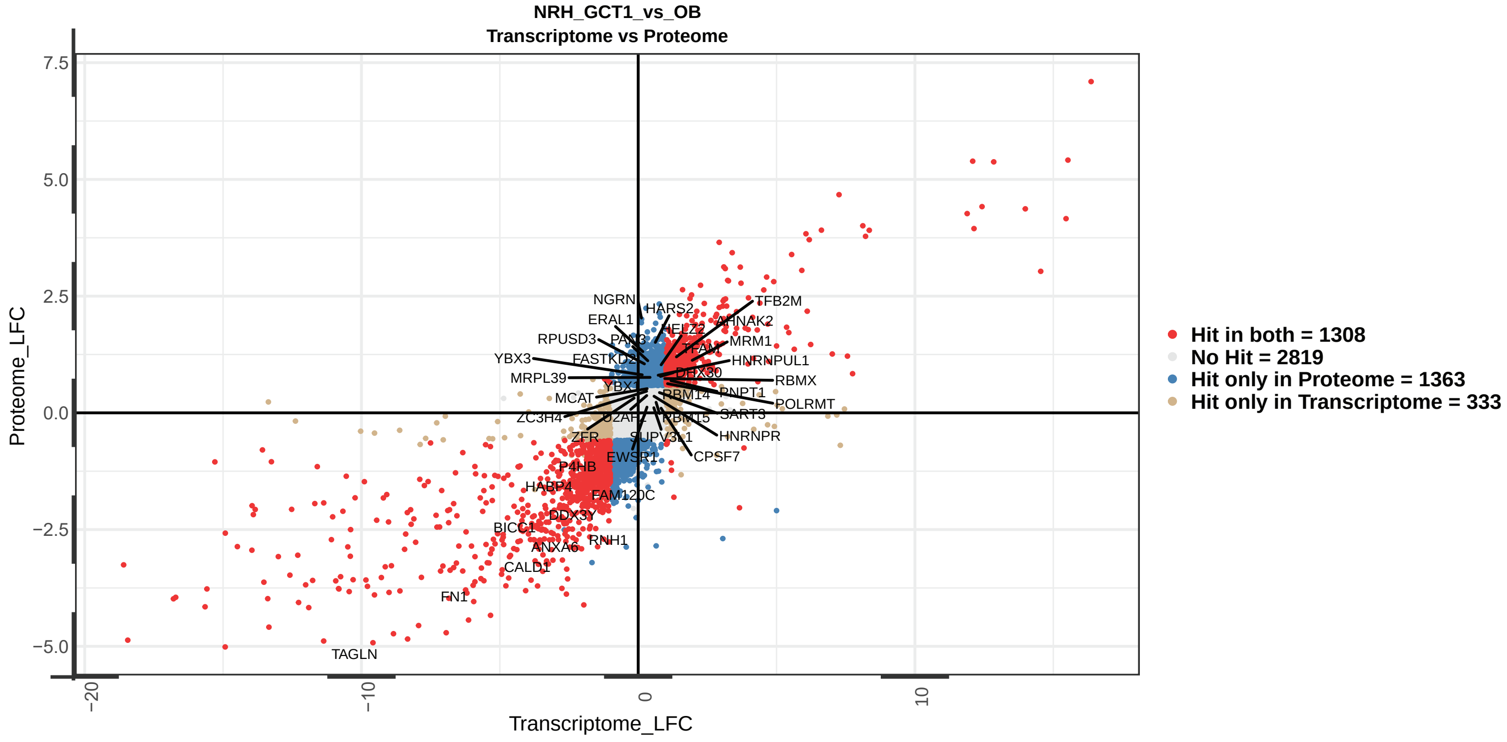
NRH_GCT1_vs_OB
Transcriptome vs Proteome
Proteome_LFC
Transcriptome_LFC
7.5
5.0
2.5
0.0
−2.5
−5.0
−20
−10
0
10
NGRN
HARS2
TFB2M
ERAL1
HELZ2
AHNAK2
RPUSD3
PAN3
MRM1
YBX3
FASTKD2
TFAM
HNRNPUL1
MRPL39
YBX1
DHX30
RBMX
MCAT
RBM14
PNPT1
POLRMT
ZC3H4
U2AF2
RBM15
SART3
ZFR
SUPV3L1
HNRNPR
EWSR1
CPSF7
P4HB
HABP4
FAM120C
DDX3Y
BICC1
RNH1
ANXA6
CALD1
FN1
TAGLN
Hit in both = 1308
No Hit = 2819
Hit only in Proteome = 1363
Hit only in Transcriptome = 333

Supplementary Figure 6. Comparison between whole transcriptome and full proteomes of OS and GCTB normalized to OB. The RNA expression levels (log2 FC) in the transcriptomes (x-axis) is plotted against the protein expression levels (log2 FC) in the full proteomes (y-axis) of the osteosarcoma and giant cell tumor of bone cells, normalized to respective expression levels in osteoblasts (OB). Hits are differentially expressed genes with adjusted p-value <0.05 and absolute log2 fold change > 1. Red dots indicate genes significantly differentially expressed in both the transcriptome and proteome, blue dots indicate genes significantly differentially expressed only in the proteome, gold dots indicate genes significantly differentially expressed only in the transcriptome and grey dots indicate genes which are unchanged in both. The top 20% RBPs enriched in the RNA interactome of each cell type are indicated in the scatter plots.

Supplementary Figure 7

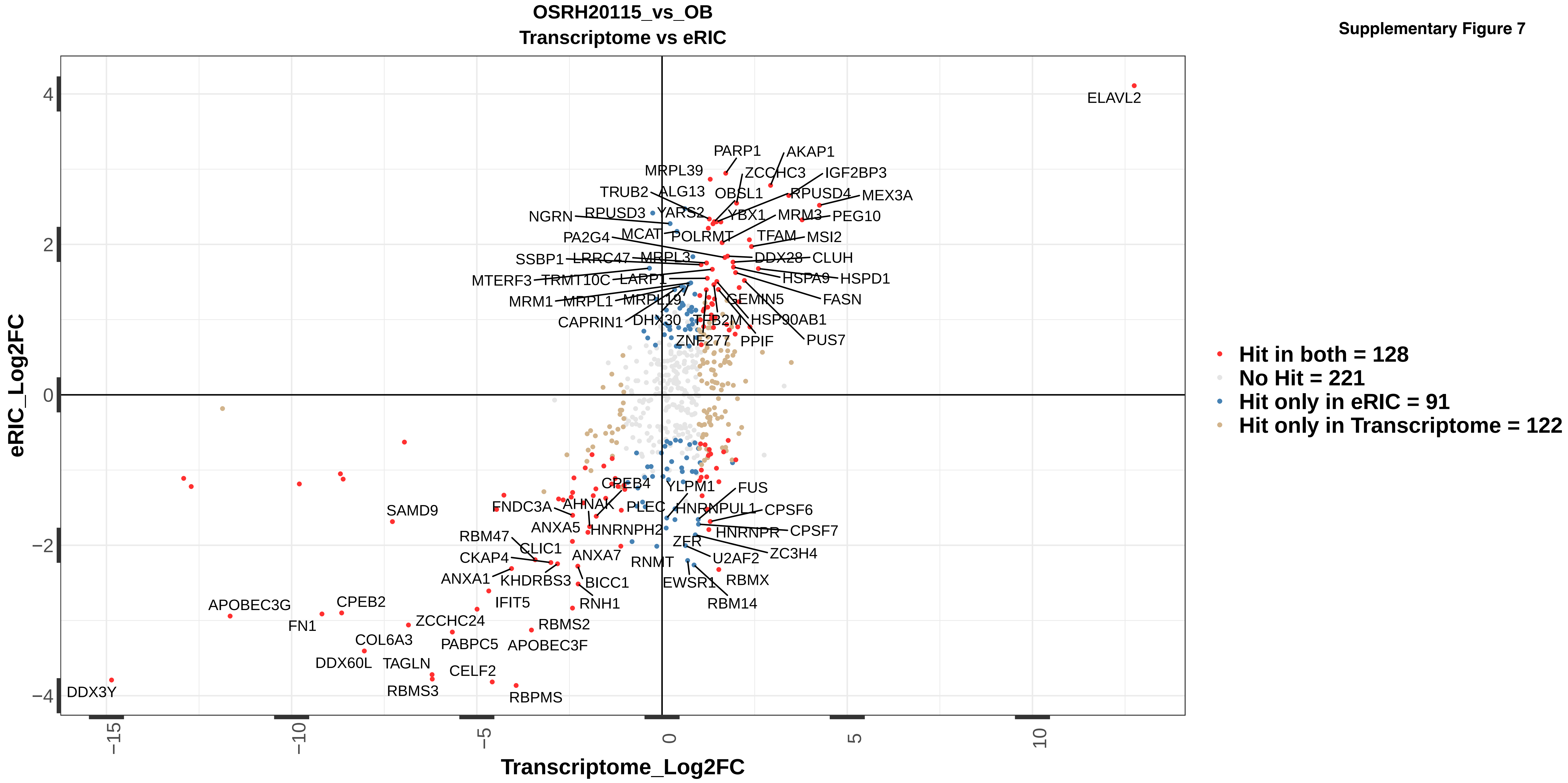

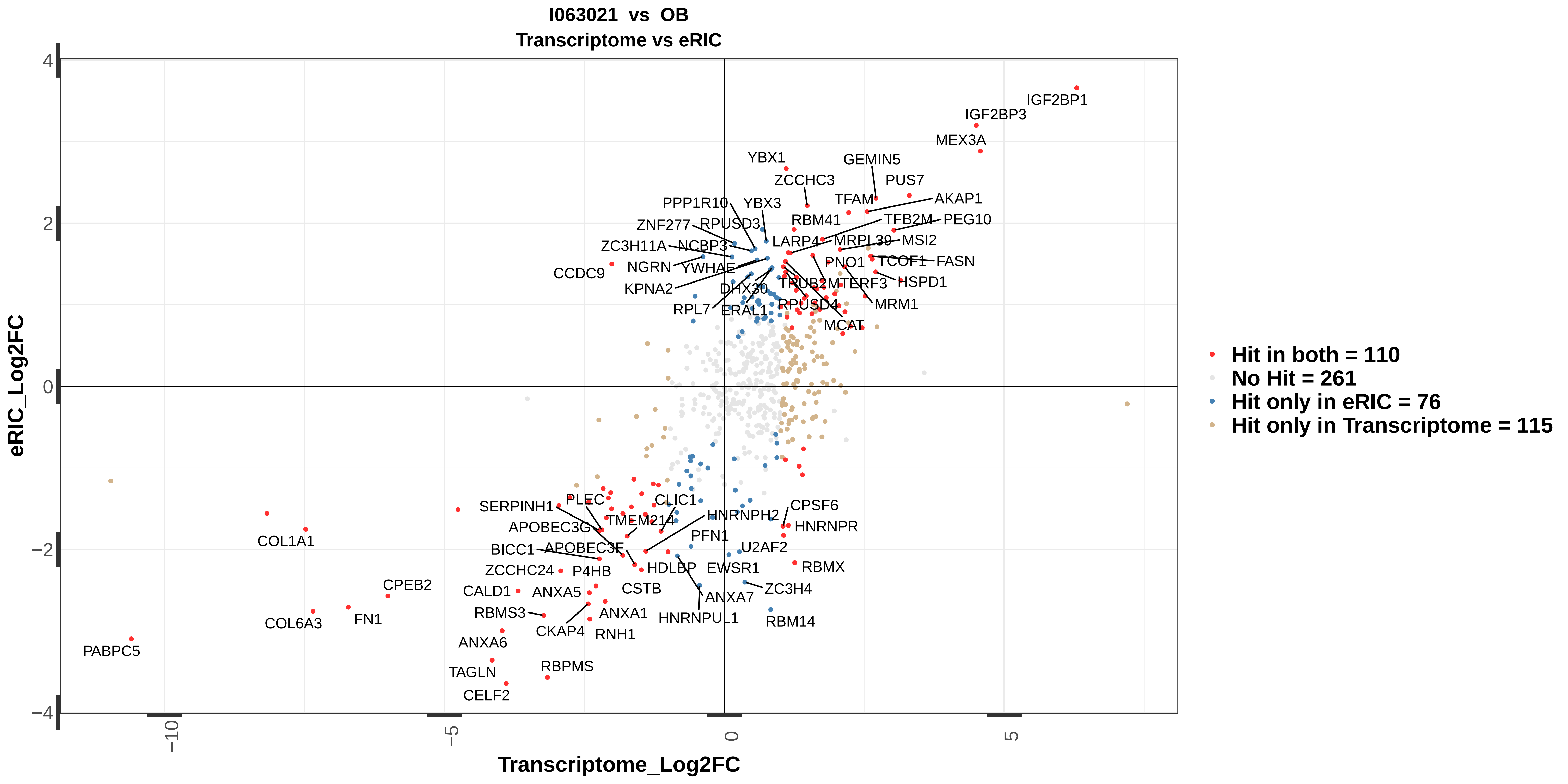

I063021_vs_OB
Transcriptome vs eRIC
eRIC_Log2FC
Transcriptome_Log2FC
Hit in both = 110
No Hit = 261
Hit only in eRIC = 76
Hit only in Transcriptome = 115
IGF2BP1
IGF2BP3
MEX3A
YBX1
GEMIN5
ZCCHC3
PUS7
TFAM
AKAP1
PPP1R10
YBX3
RBM41
TFB2M
PEG10
ZNF277
RPUSD3
LARP4
MRPL39
MSI2
ZC3H11A
NCBP3
PNO1
TCOF1
FASN
NGRN
YWHAE
CCDC9
KPNA2
DHX30
TRUB2
MTERF3
HSPD1
RPL7
ERAL1
RPUSD4
MRM1
MCAT
SERPINH1
PLEC
CLIC1
CPSF6
APOBEC3G
TMEM214
HNRNPH2
HNRNPR
COL1A1
PFN1
BICC1
APOBEC3F
U2AF2
ZCCHC24
P4HB
HDLBP
EWSR1
RBMX
CPEB2
CALD1
ANXA5
CSTB
ZC3H4
ANXA7
RBMS3
ANXA1
HNRNPUL1
RBM14
COL6A3
FN1
CKAP4
RNH1
ANXA6
PABPC5
RBPMS
TAGLN
CELF2

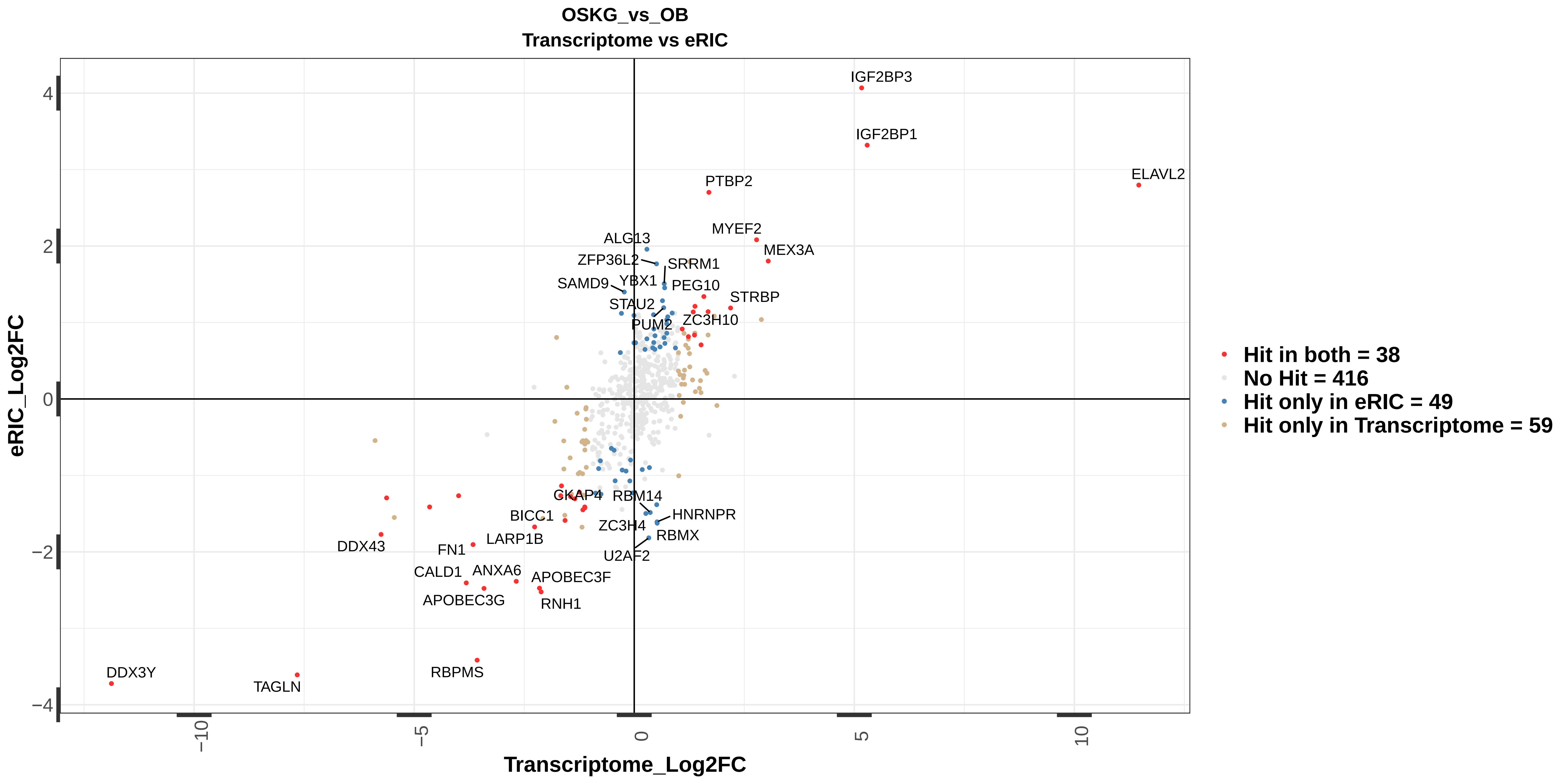

OSKG_vs_OB
Transcriptome vs eRIC
eRIC_Log2FC
Transcriptome_Log2FC
4
2
0
−2
−4
−10
−5
0
5
10
IGF2BP3
IGF2BP1
ELAVL2
PTBP2
MYEF2
MEX3A
ALG13
ZFP36L2
SRRM1
SAMD9
YBX1
PEG10
STAU2
STRBP
PUM2
ZC3H10
CKAP4
RBM14
BICC1
HNRNPR
ZC3H4
LARP1B
RBMX
DDX43
FN1
U2AF2
CALD1
ANXA6
APOBEC3F
APOBEC3G
RNH1
RBPMS
DDX3Y
TAGLN
Hit in both = 38
No Hit = 416
Hit only in eRIC = 49
Hit only in Transcriptome = 59

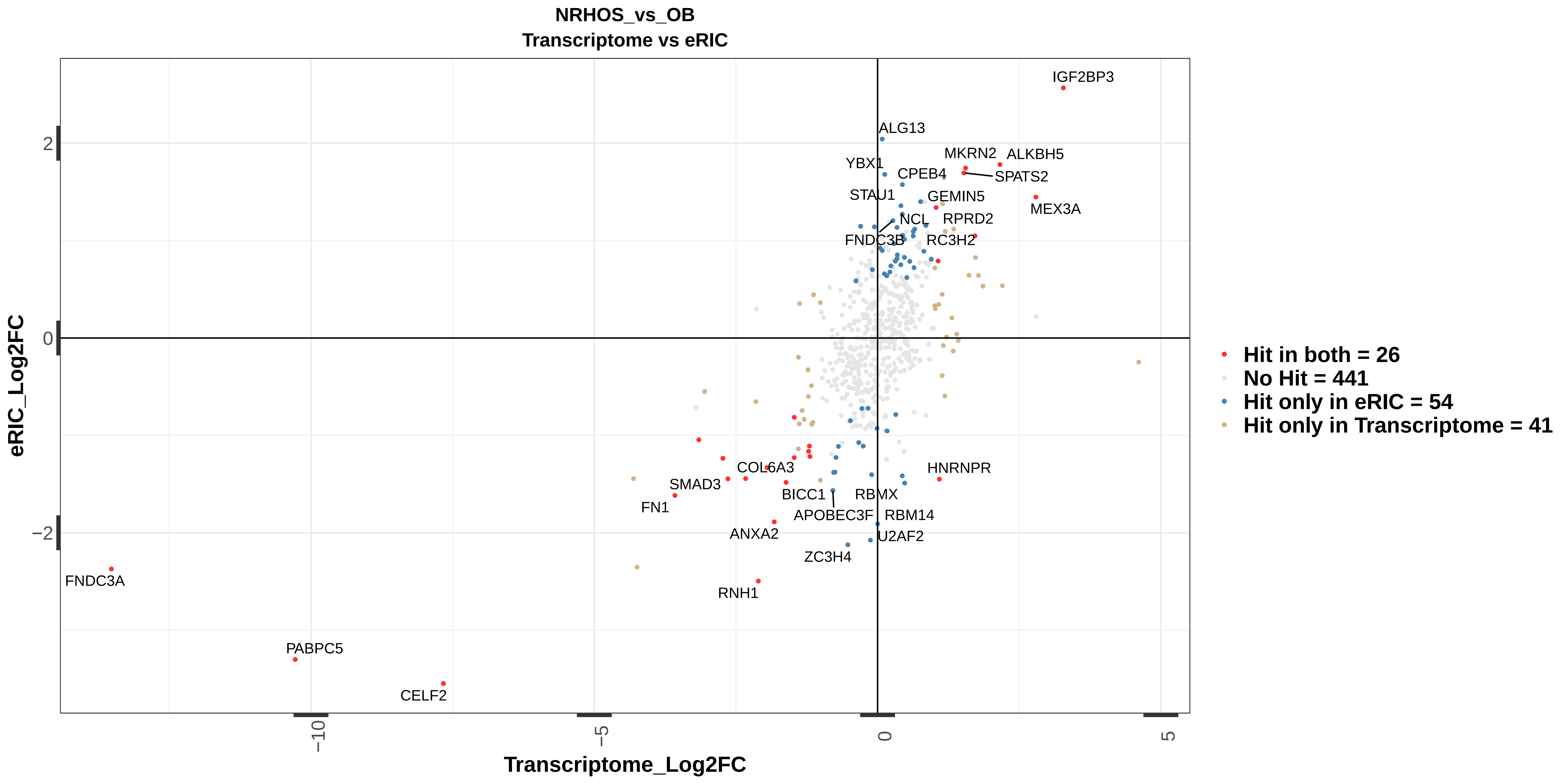

NRHOS_vs_OB
Transcriptome vs eRIC
eRIC_Log2FC
Transcriptome_Log2FC
2
0
−2
−10
−5
0
5
IGF2BP3
ALG13
MKRN2
ALKBH5
YBX1
CPEB4
SPATS2
STAU1
GEMIN5
MEX3A
NCL
RPRD2
FNDC3B
RC3H2
COL6A3
HNRNPR
SMAD3
FN1
BICC1
RBMX
APOBEC3F
RBM14
ANXA2
U2AF2
ZC3H4
FNDC3A
RNH1
PABPC5
CELF2
Hit in both = 26
No Hit = 441
Hit only in eRIC = 54
Hit only in Transcriptome = 41

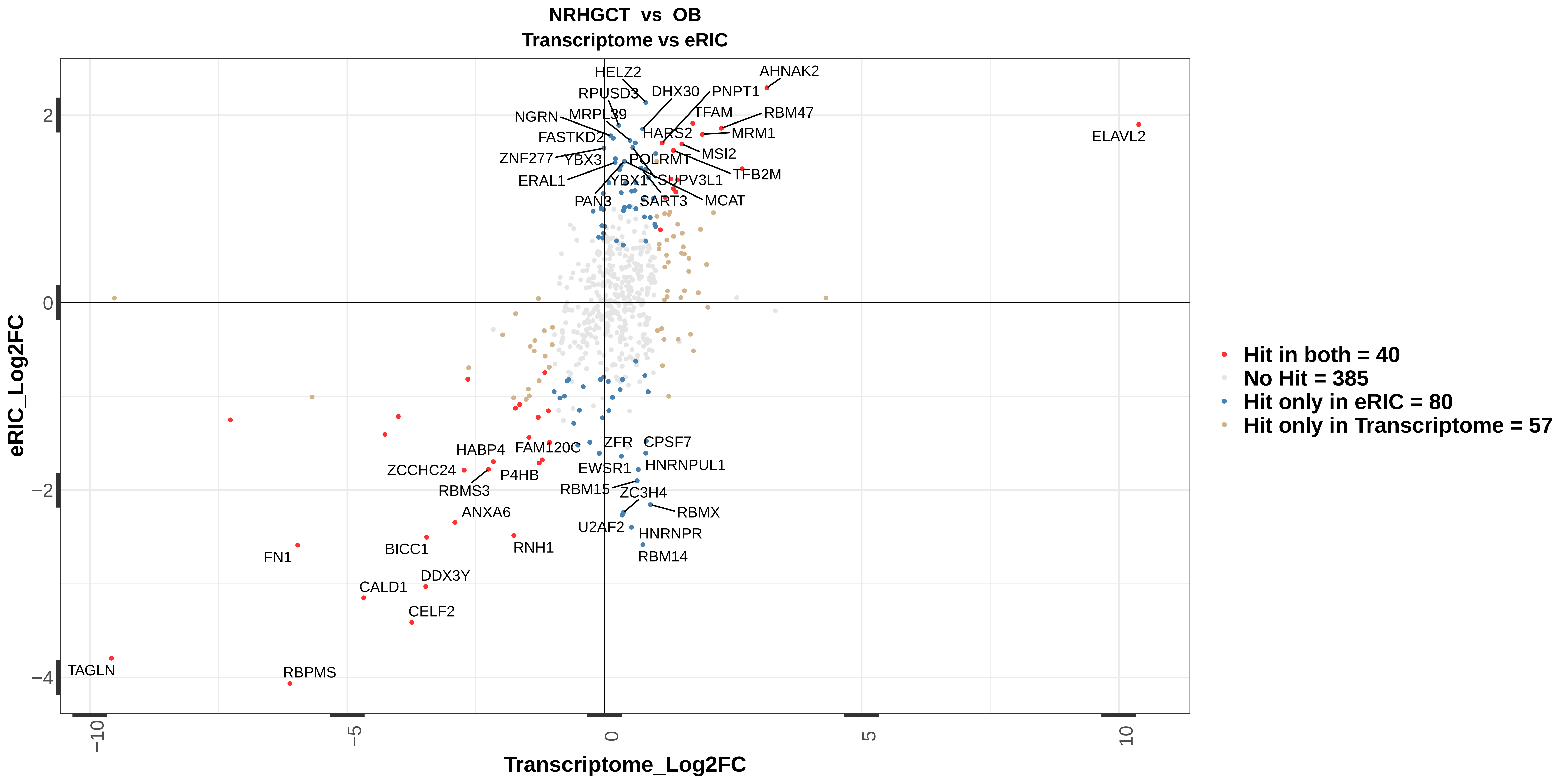
NRHGCT_vs_OB
Transcriptome vs eRIC
eRIC_Log2FC
Transcriptome_Log2FC
2
0
−2
−4
−10
−5
0
5
10
HELZ2
AHNAK2
RPUSD3
DHX30
PNPT1
NGRN
MRPL39
TFAM
RBM47
FASTKD2
HARS2
MRM1
ELAVL2
ZNF277
YBX3
POLRMT
MSI2
ERAL1
YBX1
SUPV3L1
TFB2M
PAN3
SART3
MCAT
HABP4
FAM120C
ZFR
CPSF7
ZCCHC24
P4HB
EWSR1
HNRNPUL1
RBMS3
RBM15
ZC3H4
ANXA6
RBMX
U2AF2
HNRNPR
BICC1
RNH1
RBM14
FN1
DDX3Y
CALD1
CELF2
TAGLN
RBPMS
Hit in both = 40
No Hit = 385
Hit only in eRIC = 80
Hit only in Transcriptome = 57

Supplementary Figure 7. Comparison between whole transcriptome and RNA interactome (eRIC) of OS and GCTB normalized to OB. The RNA expression levels (log2 FC) in the transcriptomes (x-axis) is plotted against the protein levels (log2 FC) in the RNA interactome captures (y-axis) of the osteosarcoma and giant cell tumor of bone cells, normalized to respective expression levels in osteoblasts (OB). Hits are differentially expressed genes with adjusted p-value <0.05 and absolute log2 fold change > 1. Red dots indicate genes significantly differentially expressed in both the transcriptome and RNA interactome, blue dots indicate genes significantly differentially expressed only in the RNA interactome, gold dots indicate genes significantly differentially expressed only in the transcriptome and grey dots indicate genes which are unchanged in both. The top 20% RBPs enriched in the RNA interactome of each cell type are indicated in the scatter plots.

Supplementary Figure 8

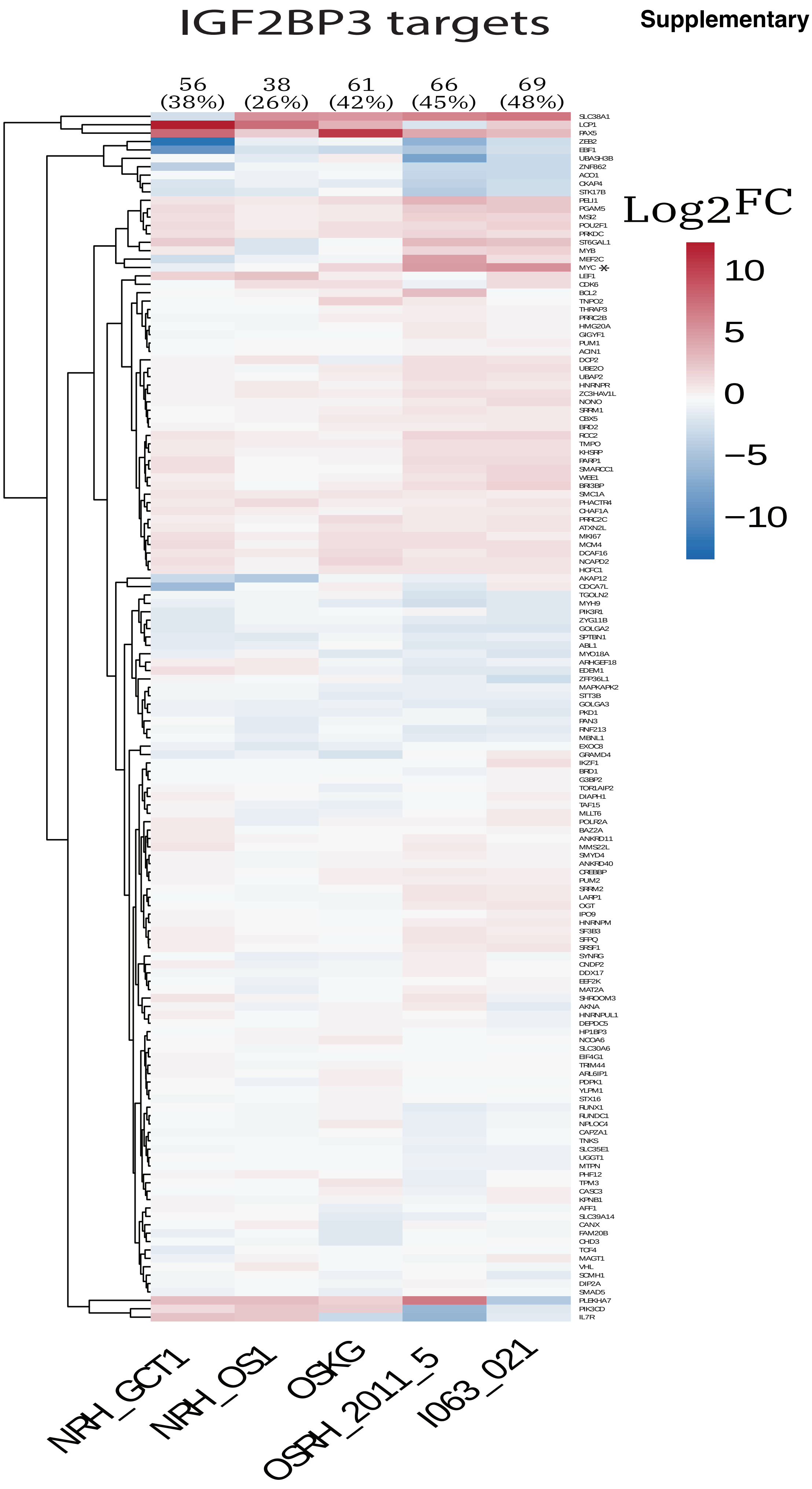

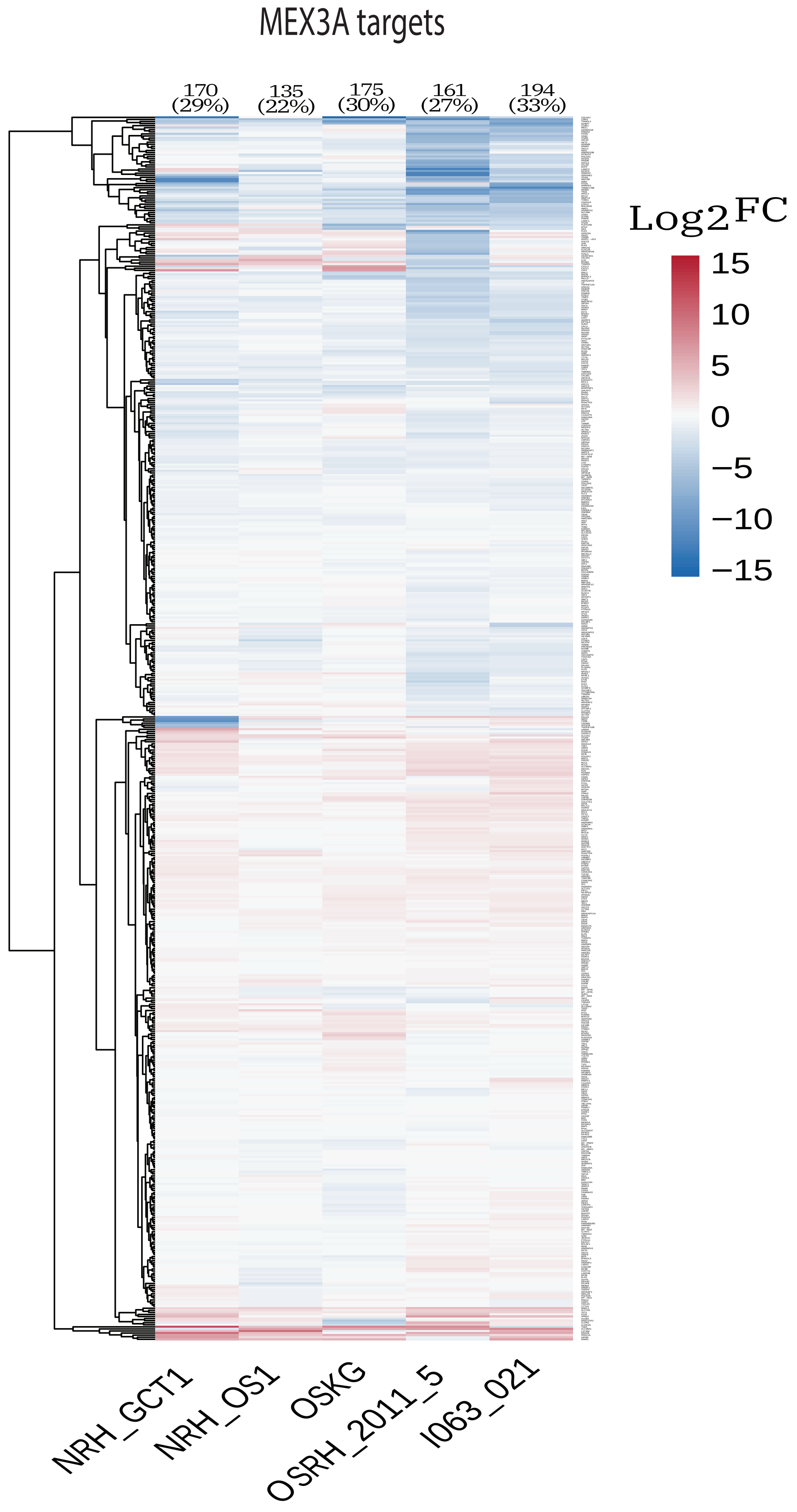

MEX3A targets
170 (29%)
135 (22%)
175 (30%)
161 (27%)
194 (33%)
Log2FC
15
10
5
0
−5
−10
−15
NRH_GCT1
NRH_OS1
OSKG
OSRH_2011_5
I063_021

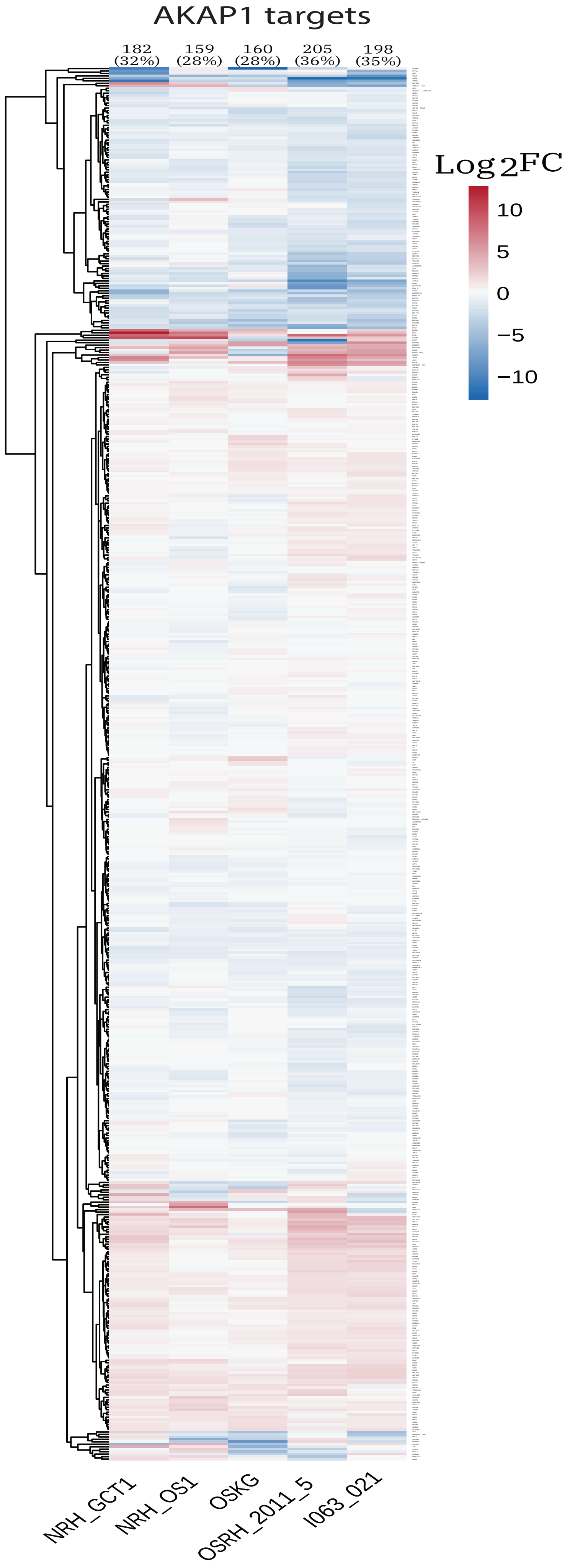
AKAP1 targets
182 (32%)
159 (28%)
160 (28%)
205 (36%)
198 (35%)
Log2FC
10
5
0
−5
−10
NRH_GCT1
NRH_OS1
OSKG
OSRH_2011_5
I063_021

Supplementary Figure 8. Target transcripts of RBPs upregulated in transcriptomes of OS and GCTB cells. Heat maps representing expression levels of target mRNAs of IGF2BP3, MEX3A and AKAP1, obtained from published eCLIP/RNAseq data (eCLIP and shRNA-mediated knockdown of IGF2BP3), RIPseq data (RIPseq for MEX3A) and eCLIP data (eCLIP of AKAP1) in the transcriptomes of the osteosarcoma and giant cell tumor of bone cells, normalized to osteoblast (OB). Genes which show differential expression (log2 FC ≥ 0.5) are indicated in the heat maps. The numbers above each column represent the absolute number and % of mRNAs showing significant difference in expression. Myc is indicated by * in the IGF2BP3 heat map.

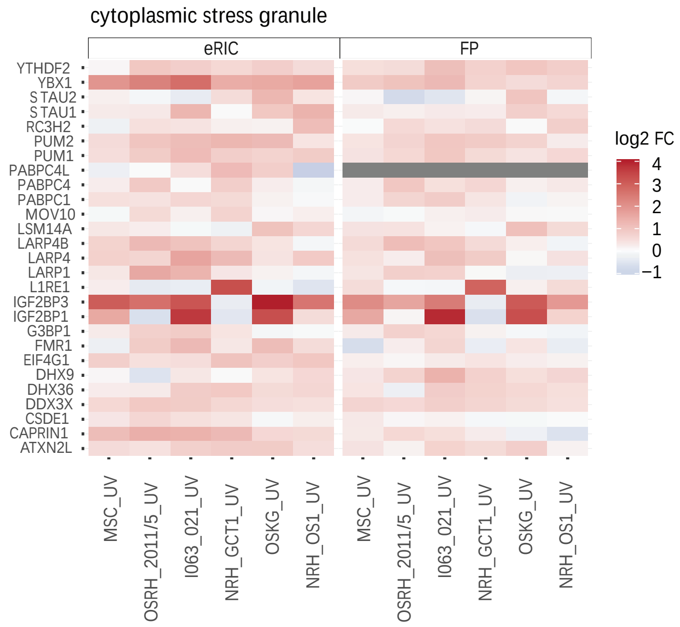

Supplementary Figure 9. Stress granule (SG) proteins are upregulated in the RNA interactomes of the sarcomata. Heatmap showing the relative RBP abundance (log2 FC) of 27 cytoplasmic stress granule proteins in comparison of sarcoma with OB either in the RNA interactome (eRIC) and in the full proteome (FP).
