# Supplementary material for: Systematic analysis of RNA-binding proteins identifies targetable therapeutic vulnerabilities in osteosarcoma
Source: Nat Commun. 2024 Apr 1;15:2810. doi: 10.1038/s41467-024-47031-y (PMC10984982; doi:10.1038/s41467-024-47031-y)
Supplement: Supplementary file 12 — Reporting Summary [file 41467_2024_47031_MOESM12_ESM.pdf]

nature portfolio

Corresponding author(s): Dr. Andreas E. Kulozik
Dr. Matthias W. Hentze

Last updated by author(s): Mar 1, 2024

Reporting Summary

Nature Portfolio wishes to improve the reproducibility of the work that we publish. This form provides structure for consistency and transparency in reporting. For further information on Nature Portfolio policies, see our Editorial Policies and the Editorial Policy Checklist.

Statistics

For all statistical analyses, confirm that the following items are present in the figure legend, table legend, main text, or Methods section.

n/a	Confirmed	
☐	☒	The exact sample size (*n*) for each experimental group/condition, given as a discrete number and unit of measurement
☐	☒	A statement on whether measurements were taken from distinct samples or whether the same sample was measured repeatedly
☐	☒	The statistical test(s) used AND whether they are one- or two-sided<br>*Only common tests should be described solely by name; describe more complex techniques in the Methods section.*
☒	☐	A description of all covariates tested
☐	☒	A description of any assumptions or corrections, such as tests of normality and adjustment for multiple comparisons
☐	☒	A full description of the statistical parameters including central tendency (e.g. means) or other basic estimates (e.g. regression coefficient) AND variation (e.g. standard deviation) or associated estimates of uncertainty (e.g. confidence intervals)
☐	☒	For null hypothesis testing, the test statistic (e.g. *F*, *t*, *r*) with confidence intervals, effect sizes, degrees of freedom and *P* value noted<br>*Give P values as exact values whenever suitable.*
☒	☐	For Bayesian analysis, information on the choice of priors and Markov chain Monte Carlo settings
☒	☐	For hierarchical and complex designs, identification of the appropriate level for tests and full reporting of outcomes
☒	☐	Estimates of effect sizes (e.g. Cohen's *d*, Pearson's *r*), indicating how they were calculated

Our web collection on statistics for biologists contains articles on many of the points above.

Software and code

Policy information about availability of computer code

Data collection	Mass spectrometry (MS) data was collected using an Orbitrap Fusion™ Lumos™ Tribrid™ Mass Spectrometer (Thermo Fischer Scientific). The acquired MS data was processed using IsobarQuant (Franken et al., 2015) and Mascot (v2.2.07). Data were searched against the human Uniprot proteome database (UP000005640).<br>The copy number variations of genomic segments were inferred from the methylation array (Infinium MethylationEPIC BeadChip microarray) data based on the R-package conumee after additional baseline correction (https://github.com/dstichel/conumee).<br>RNAseq data was obtained from Illumina NextSeq 2000 platform with a 100 paired end sequencing program on a P2 flowcell. The raw reads were trimmed using Cutadapt (v4.4)97 and aligned to the human genome (GRCh38.p13) with STAR (v2.7.10.b)98. Aligned reads were summarized with featureCounts (v2.0.6)99. DESeq2 (v1.40.2)100 using local dispersion fit and the Wald test with IHW101 for multiple hypothesis correction was used to determine significantly differentially regulated genes in each test sample versus control samples comparison,
Data analysis	R programming language (ISBN 3-900051-07-0)<br>R (v 4.0.4)<br>packages:<br>limma (Ritchie et al., 2015), https://bioconductor.org/packages/limma/<br>vsn (Huber et al., 2002), https://bioconductor.org/packages/vsn/<br>MSnbase (Gatto et al., 2012), https://bioconductor.org/packages/MSnbase/<br>tidyverse (Wickham et al., 2019), https://tidyverse.tidyverse.org/<br>biobroom (Bass et al., 2015), https://bioconductor.org/packages/biobroom/<br>ggrepel (Slowikowski et al., 2018), https://cran.r-project.org/web/packages/ggrepel/vignettes/ggrepel.html

ggpubr (Kassambara, 2016), https://CRAN.R-project.org/package=ggpubr
clusterProfiler (Yu, et al., 2012), https://bioconductor.org/packages/clusterProfiler/
org.Hs.eg.db (Carlson, 2016), https://bioconductor.org/packages/org.Hs.eg.db/
ConsensusClusterPlus (Wilkersonet al., 2010), https://bioconductor.org/packages/ConsensusClusterPlus/
msigdbr (Dolgalev, 2018), https://igordot.github.io/msigdbr/
UpSetR (Conway et al., 2017), https://github.com/cran/UpSetR
ggdendro (de Vries et al., 2014), https://CRAN.R-project.org/package=ggdendro
others:
RBPbase (https://rbpbase.shiny.embl.de, v.0.2.0)
DAVID (https://david.ncifcrf.gov/tools.jsp, v.6.8)
DescribePROT database (Zhao et al., 2021)
For analysis of RNAseq data raw reads were trimmed using Cutadapt (v4.4) and aligned to the human genome (GRCh38.p13) with STAR (v2.7.10.b). Aligned reads were summarized with featureCounts (v2.0.6). DESeq2 (v1.40.2)100 using local dispersion fit and the Wald test with IHW101 for multiple hypothesis correction was used to determine significantly differentially regulated genes in each test sample versus control samples comparison

For manuscripts utilizing custom algorithms or software that are central to the research but not yet described in published literature, software must be made available to editors and reviewers. We strongly encourage code deposition in a community repository (e.g. GitHub). See the Nature Portfolio guidelines for submitting code & software for further information.

Data

Policy information about availability of data

All manuscripts must include a data availability statement. This statement should provide the following information, where applicable:

- Accession codes, unique identifiers, or web links for publicly available datasets
- A description of any restrictions on data availability
- For clinical datasets or third party data, please ensure that the statement adheres to our policy

The mass spectrometry proteomics data have been deposited to the ProteomeXchange Consortium via the PRIDE105 partner repository with the dataset identifier PXD038185 (http://www.ebi.ac.uk/pride/archive/projects/PXD038185). The RNAseq transcriptomic data have been deposited in NCBI's Gene Expression Omnibus106 and are accessible through GEO Series accession number GSE246405 (https://www.ncbi.nlm.nih.gov/geo/query/acc.cgi?acc=GSE246405). The transcriptomic, eCLIPseq and RIPseq datasets used to generate Supplementary Figure 8 and Supplementary Data 8 are publicly available as supplementary data in Palanichamy et al., Qiu et al. and in the ENCODE database (https://www.encodeproject.org/genes/8165/). The processed complete eRIC, full proteome and transcriptome data is available as Supplementary Data 9 and 10. The remaining data are available within the Article, Supplementary Information or Source Data files.

Human research participants

Policy information about studies involving human research participants and Sex and Gender in Research.

Reporting on sex and gender	The material of the 5 patients analyzed here were included based on diagnosis and availability of suitable material. Of the 5 patients 1 were female and 4 were male. Sex and/or gender was of the patients were not considered in the study design. Sex- and gender-based analysis was considered not relevant to the study. At the time of diagnosis the 5 patients analyzed here were 12-, 14-, 14-, 24- and 31-years old.
Population characteristics	*Describe the covariate-relevant population characteristics of the human research participants (e.g. age, genotypic information, past and current diagnosis and treatment categories). If you filled out the behavioural & social sciences study design questions and have nothing to add here, write "See above."*
Recruitment	The material of the patients analyzed here were included into the current study on the basis of the diagnosis of a malignant bone tumor, availability of material and approval of the respective ethics committees for the use of these samples. Although there was no active bias in including material, the sample size does, of course, limit to generalize the proof-of-principle findings reported here to a larger group of osteosarcoma patients.
Ethics oversight	The use of samples OSRH, OSKG and IO63_021 was approved by the ethics committee of Medical Faculty of Heidelberg University, Germany. The use of samples NRH_GCT1 and NRH_OS1 was approved by the Committee for Ethics Southeastern Norway. Written informed consent of the patients and their guardians were obtained.

Note that full information on the approval of the study protocol must also be provided in the manuscript.

Field-specific reporting

Please select the one below that is the best fit for your research. If you are not sure, read the appropriate sections before making your selection.

☒ Life sciences ☐ Behavioural & social sciences ☐ Ecological, evolutionary & environmental sciences

For a reference copy of the document with all sections, see nature.com/documents/nr-reporting-summary-flat.pdf

Life sciences study design

All studies must disclose on these points even when the disclosure is negative.

Sample size	Sample size for enhanced RNA interactome capture (eRIC), proteomic and transcriptomic analyses was determined by the availability of primary cell samples derived from osteosarcoma, the most common type of bone cancer in children and adolescents, but a rare disease in general. No sample size calculations were performed as all accessible cases were included and the obtained RNA interactome data largely resembles typical features of RNA interactomes published for other origins, and both sufficient similarity and significant differences were observed statistically for different individuals.
Data exclusions	Mass spectrometry data were filtered for common laboratory contaminants and reversed sequences. Proteins with less than two quantified unique peptides matched and proteins detected only in one of the two biological replicates were removed for analyses.
Replication	All experiments were performed in at least 2 biological replicates as reported in the figure legends and method section, the findings were consistently reproduced.
Randomization	Randomization was not applicable for patient sample chosen as we accessed all sample cases available to us. Randomization was not necessary for mass spectrometry (MS) experiment, as the library preparation (either eRIC or full proteome) was performed by multiplexing different samples using TMT isobaric labeling and therefore all samples were simultaneously analyzed in the same MS run for each biological replicate. There were no interventions to randomize in other experiments in this study.
Blinding	Blinding was not possible for the analysis of sarcoma sample data compared to the control cells, but subgroups of sarcoma samples were objectively determined by quantitative differences of RNA interactome data.

Reporting for specific materials, systems and methods

We require information from authors about some types of materials, experimental systems and methods used in many studies. Here, indicate whether each material, system or method listed is relevant to your study. If you are not sure if a list item applies to your research, read the appropriate section before selecting a response.

Materials & experimental systems

n/a	Involved in the study	
☐	☒	Antibodies
☐	☒	Eukaryotic cell lines
☒	☐	Palaeontology and archaeology
☒	☐	Animals and other organisms
☐	☒	Clinical data
☒	☐	Dual use research of concern

Methods

n/a	Involved in the study	
☒	☐	ChIP-seq
☒	☐	Flow cytometry
☒	☐	MRI-based neuroimaging

Antibodies

Antibodies used	CSDE1 (#13319-1-AP, Proteintech) (1:4000 dilution)<br>HuR (#11910-1-AP, Proteintech) (1:5000 dilution)<br>hnRNPK (#11426-1-AP, Proteintech) (1:5000 dilution)<br>Histone H3 (#9715S, Cell Signaling Technology) (1:5000 dilution)<br>α-tubulin (#T5168, Merck) (1:4000 dilution)<br>β-actin (#A1978, Merck) (1:4000 dilution)<br>Myc (#10828-1-AP, Proteintech) (1: 3000 dilution)<br>IGF2BP3 (#14642-1-AP, Proteintech) (1:5000 dilution)<br>anti-mouse (#A9044, Merck) (1:10000 dilution)<br>anti-rabbit (#A0545, Merck) (1: 10000 dilution)
Validation	Antibodies were validated by the manufacturer and other published applications, and the relevant information is provided in the manufacturer's website.<br>CSDE1 : https://www.ptglab.com/products/CSDE1-Antibody-13319-1-AP.htm<br>HuR: https://www.ptglab.com/products/HuR-Antibody-11910-1-AP.htm<br>hnRNPK: https://www.ptglab.com/products/HNRNPK-Antibody-11426-1-AP.htm<br>Histone H3 : https://www.cellsignal.com/products/primary-antibodies/histone-h3-antibody/9715<br>α-tubulin: https://www.sigmaaldrich.cn/CN/en/product/sigma/t5168<br>β-actin: https://www.sigmaaldrich.cn/CN/en/product/sigma/a1978<br>Myc: https://www.ptglab.com/products/MYC-Antibody-10828-1-AP.htm<br>IGF2BP3: https://www.ptglab.com/products/IGF2BP3-Antibody-14642-1-AP.htm

Eukaryotic cell lines

Policy information about cell lines and Sex and Gender in Research

Cell line source(s)	Primary tumor cells derived from 4 osteosarcoma patients (OSRH_2011/5, OSKG, I063_021 and NRH_OS1) and 1 giant cell tumor of bone patient (NRH_GCT1).<br>The OSRH_2011/5 (female) cells were obtained from the tumor tissue grown in an orthotopic xenotransplanted mouse model from the relapsed tumor as previously described by Dr. Blattmann (Blattmann, C. et al., 2015).<br>The OSKG (male) and I063_021 (male) cells were generated directly from the clinical biopsies from patients treated in Germany.<br>The NRH_GCT1 (male) and NRH_OS1 (male) cells were generated from tumor tissues grown in xenotransplanted mouse models and provided by Dr. Ola Myklebost (Oslo, Norway).<br>The osteoblasts (OB, C-12720, PromoCell) and bone marrow derived mesenchymal stem cells (MSCs, C-12974, PromoCell) were purchased from PromoCell, Germany.
Authentication	Tumor cells were authenticated using either CytoSNP or STR, and all cells were subjected to sarcoma classification (Koelsche, C. et al., 2021) based on DNA methylation array (Infinium MethylationEPIC BeadChip microarray) data. All cells derived from the 4 osteosarcoma tumors were predicted as high-grade osteosarcoma, and the cells derived from giant cell tumor of bone ((NRH_GCT1) were predicted as giant cell tumor of bone by the sarcoma classifier.
Mycoplasma contamination	All cells were regularly tested for mycoplasma and were free from contamination.
Commonly misidentified lines (See ICLAC register)	No commonly misidentified cell lines were used.

Clinical data

Policy information about clinical studies

All manuscripts should comply with the ICMJEguidelines for publication of clinical research and a completedCONSORT checklist must be included with all submissions.

Clinical trial registration	No clinical trial was performed.
Study protocol	Does not apply.
Data collection	Patients from whom primary tumour material was obtained underwent standard, state-of-the-art clinical care and outcomes were recorded. The clinical data were obtained according to the Helsinki declaration after informed consent has been obtained.
Outcomes	No clinical trial outcomes to report.
