# Supplementary material for: Systematic analysis of RNA-binding proteins identifies targetable therapeutic vulnerabilities in osteosarcoma
Source: Nat Commun. 2024 Apr 1;15:2810. doi: 10.1038/s41467-024-47031-y (PMC10984982; doi:10.1038/s41467-024-47031-y)
Supplement: Supplementary file 13 — Description of Additional Supplementary Files [file 41467_2024_47031_MOESM13_ESM.pdf]

Title: Supplementary Data 1.
Description: Protein idenfificafion and list of RBPs idenfified in eRIC. Proteins significantly enriched in the UV sample compared to the noUV control with a FC ≥ 2 and an FDR < 0.05 were classified as high probability RBPs ("hits") in eRIC, related to Figure 2.

Title: Supplementary Data 2.
Description: Gene ontology enrichment analyses and PFAM domain analysis of RBPs idenfified in eRIC. Related to Figure 2d, 2f, 5d.

Title: Supplementary Data 3.
Description: RBPs idenfified in different poly(A) RNA interactomes, their known RNA-binding domains and enzymafic funcfions. Related to Figure 2e, Supplementary Figure 2.

Title: Supplementary Data 4.
Description: Comparison of RBPs from RNA interactomes. Significantly altered RBPs (FDR < 0.05 and FC ≥ 1.5 in the UV versus UV sample comparisons in the eRIC) were classified as "hits". Related to Figure 4.

Title: Supplementary Data 5.
Description: Comparison between whole transcriptome and full proteome of OS and GCTB normalized to OB. Genes significantly altered in the transcriptome (adjusted p-value ≤0.05; absolute FC ≥ 2) and in the proteome (adjusted p-value ≤0.05; absolute FC ≥ 2) were classified as "hits". Related to Supplementary Figure 3,4.

Title: Supplementary Data 6. Genes upregulated in proteomes but not in transcriptomes of OS and
Description: GCTB normalized to OB. Genes significantly upregulated in the proteome (adjusted p-value ≤0.05; absolute FC ≥ 2) but not in the transcriptome are included. Related to Supplementary Figure 6.

Title: Supplementary Data 7.
Description: Correlation between RNA-binding activity (as defined by protein abundance detected in eRIC) and total protein abundance in the full proteome (FP). Related to Figure 4.

Title: Supplementary Data 8.
Description: Intersection set of genes that are iCLIPseq targets of IGF2BP3 and are downregulated (FC ≤ 1.5) upon depletion of IGF2BP3 in RS4;11 cells (analyzed from datasets in Palanichamy et al.52). Related to Supplementary Figure 8.

Title: Supplementary Data 9.
Description: Complete processed data of full proteome and RNA interactome capture analysis.

Title: Supplementary Data 10.
Description: Complete processed data of whole transcriptome analysis.
