# Supplementary material for: Systematic analysis of RNA-binding proteins identifies targetable therapeutic vulnerabilities in osteosarcoma
Source: Nat Commun. 2024 Apr 1;15:2810. doi: 10.1038/s41467-024-47031-y (PMC10984982; doi:10.1038/s41467-024-47031-y)
Supplement: Supplementary file 15 — Source Data [file 41467_2024_47031_MOESM15_ESM.zip › Source data for blots.pdf]

Figure 1 c

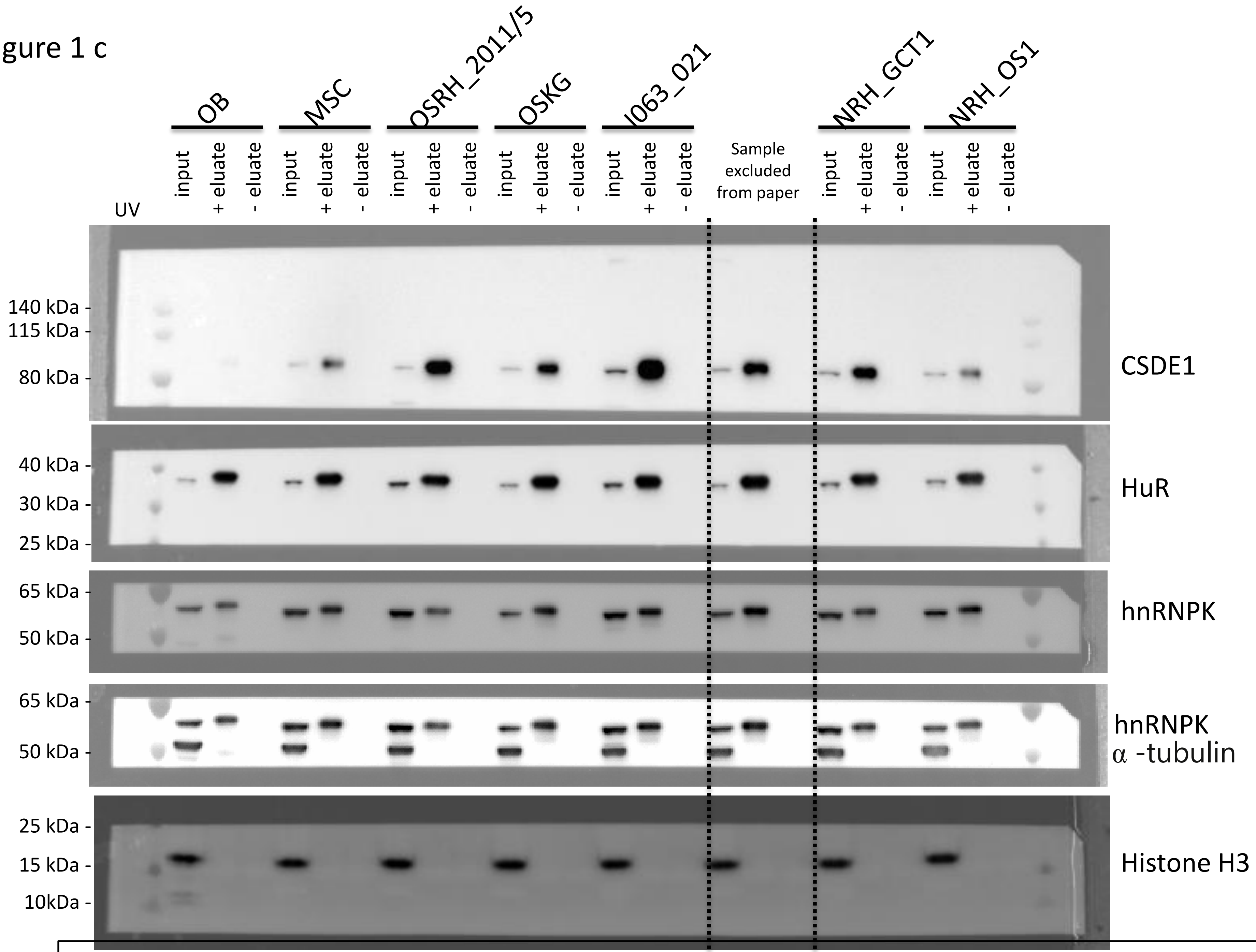

Note: α –tubulin was blotted in the same membrane after hnRNPK was blotted.

Figure 8 c

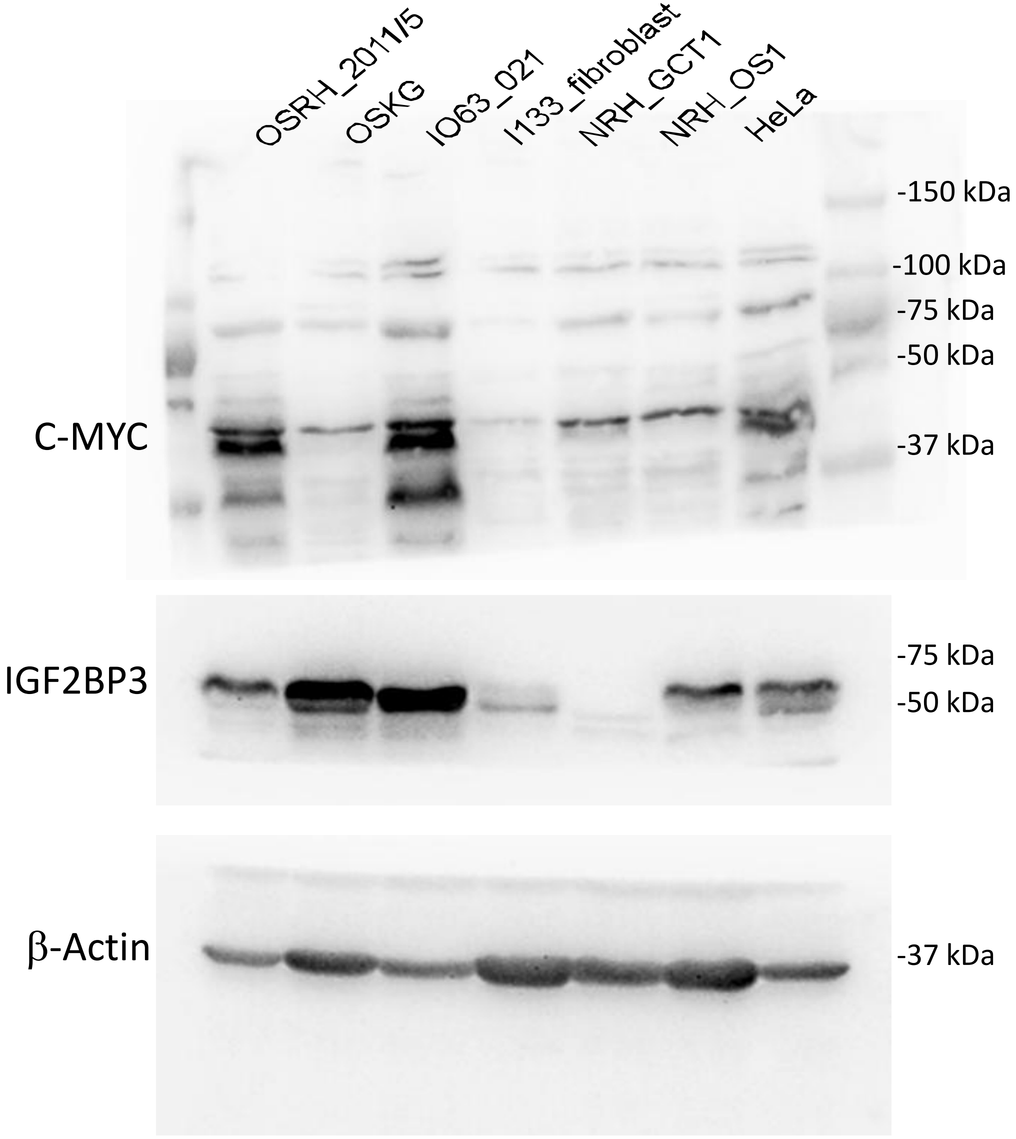

Note: C-MYC was blotted from one blot and IGF2BP3 and b-Actin was blotted from another blot run in parallel, with the same cell lysates.

Figure 8 d

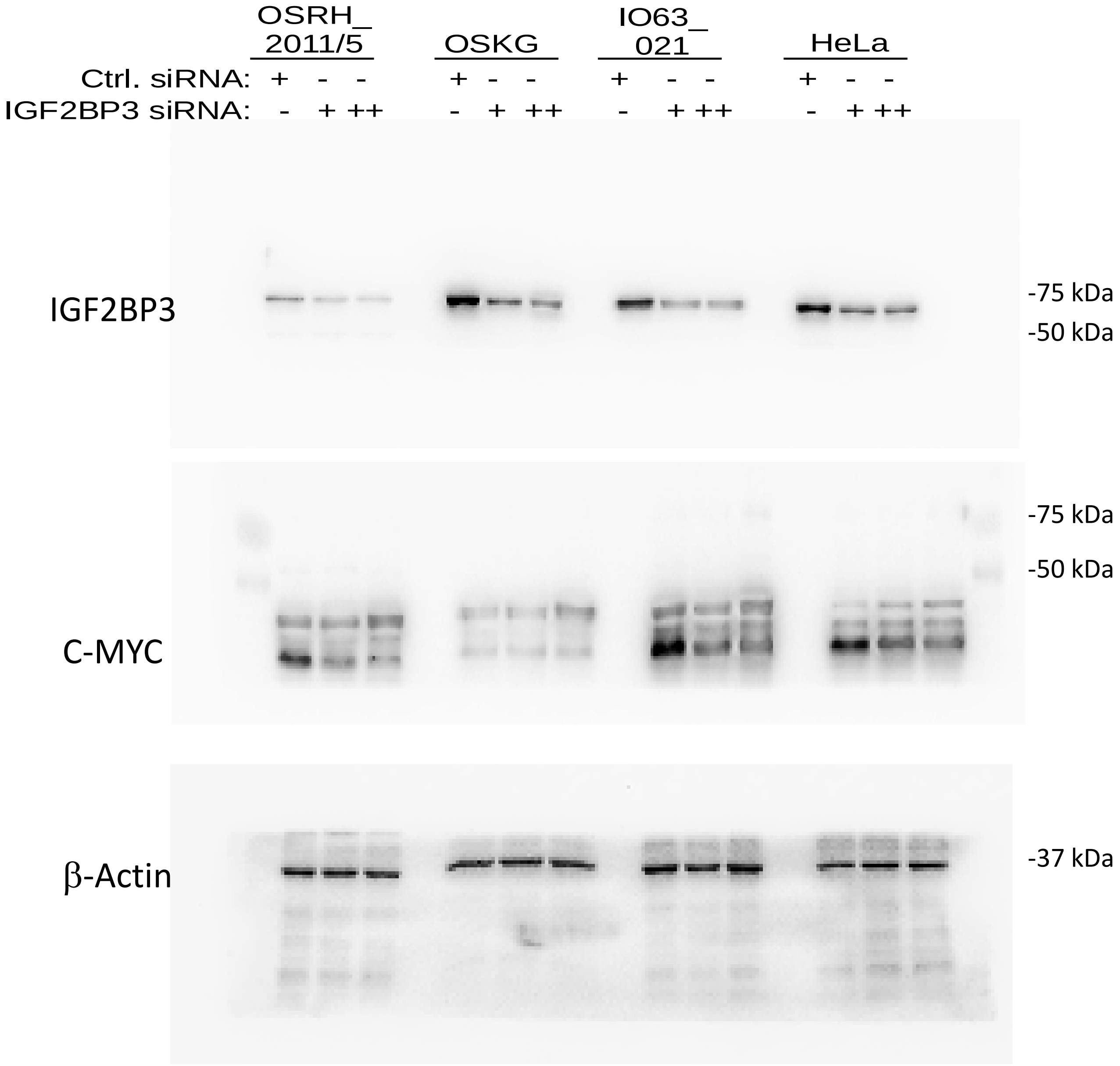

Note: C-MYC was blotted from one blot and IGF2BP3 and b-Actin was blotted from another blot run in parallel, with the same cell lysates.

Figure 8 f

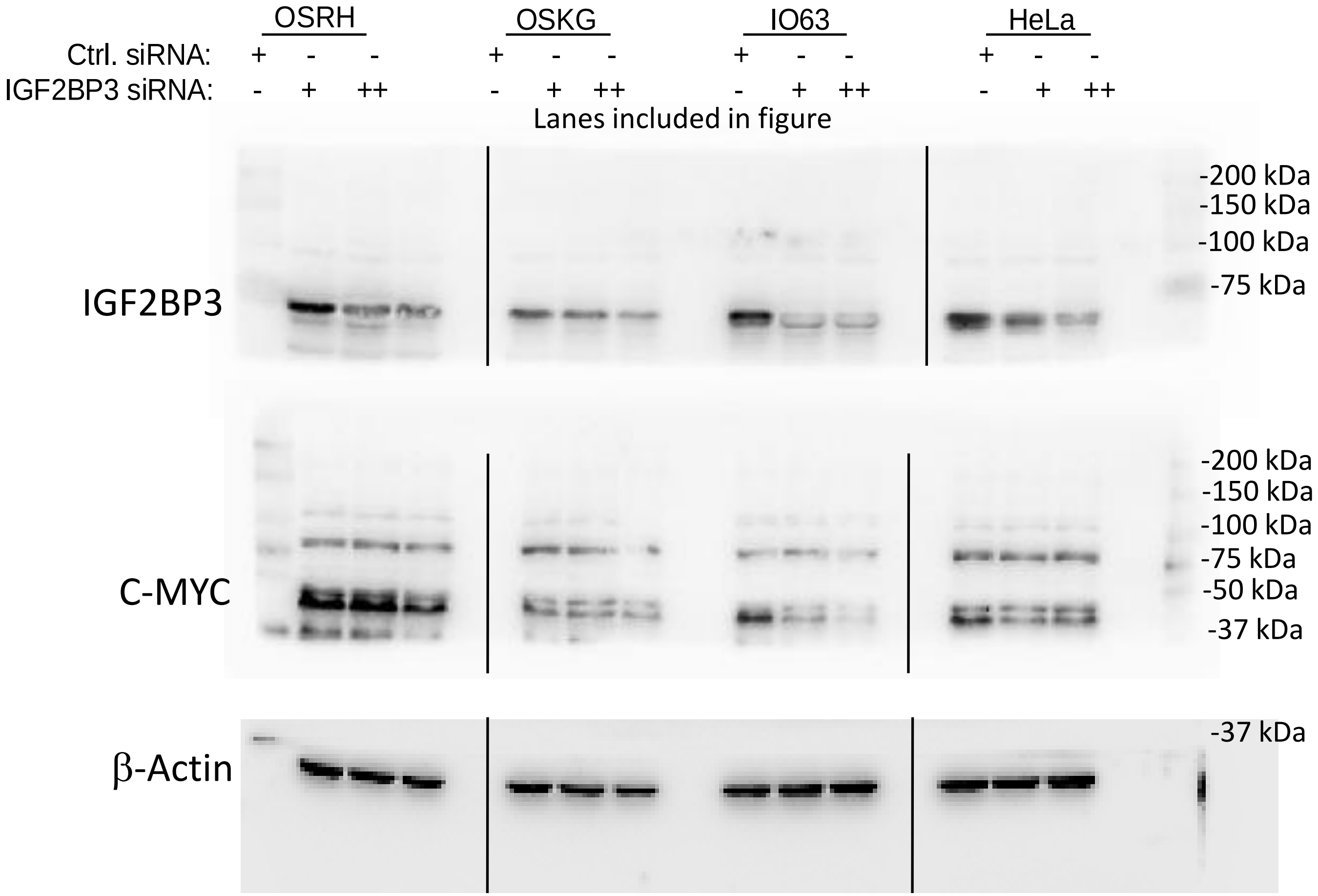

Note: C-MYC was blotted from one blot and IGF2BP3 and b-Actin was blotted from another blot run in parallel, with the same cell lysates.

Figure 8 g

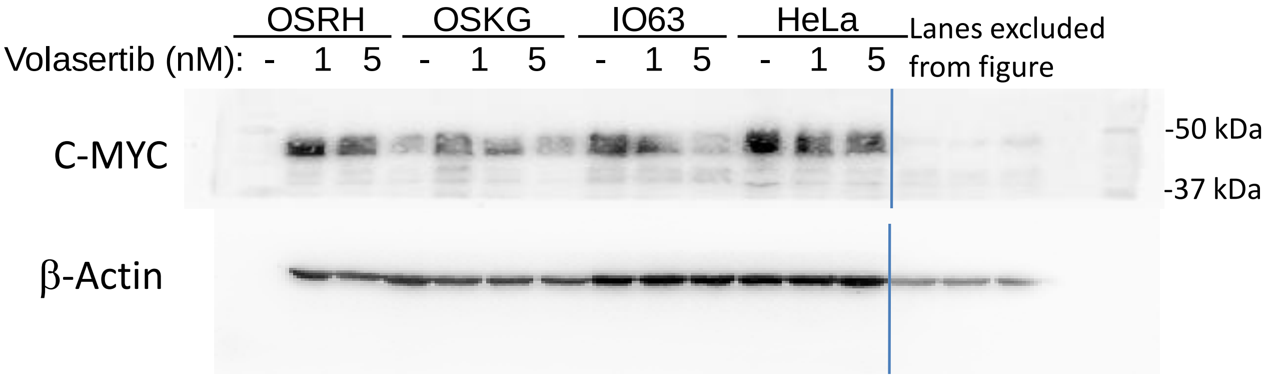

OSRH
OSKG
IO63
HeLa
Lanes excluded from figure
Volasertib (nM): - 1 5 - 1 5 - 1 5 - 1 5
C-MYC
β-Actin
-50 kDa
-37 kDa

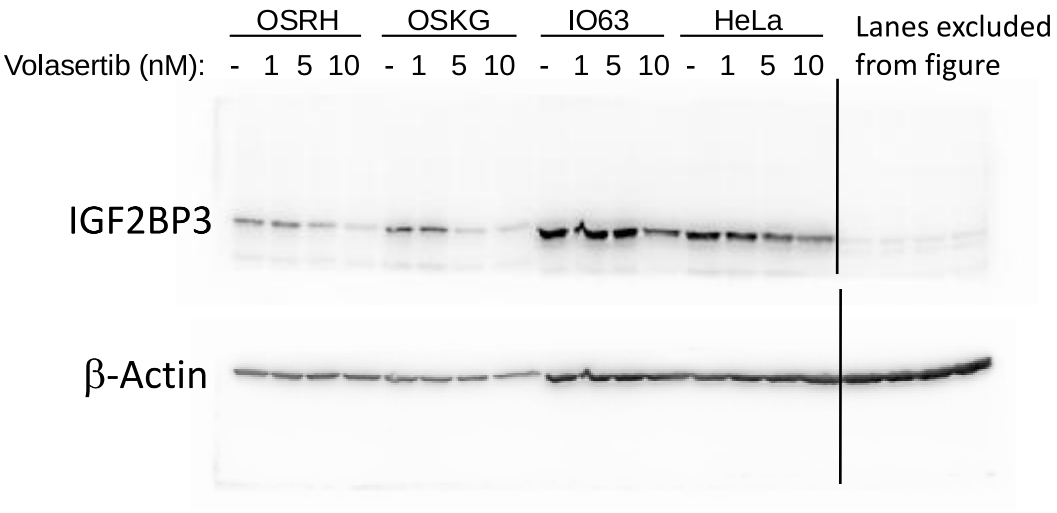

OSRH
OSKG
IO63
HeLa
Lanes excluded from figure
Volasertib (nM): - 1 5 10 - 1 5 10 - 1 5 10 - 1 5 10
IGF2BP3
β-Actin
